# Presence of Different Ceramide Species Modulates Barrier Function and Structure of Stratum Corneum Lipid Membranes: Insights from Molecular Dynamics Simulations

**DOI:** 10.1021/acs.molpharmaceut.5c00580

**Published:** 2025-06-25

**Authors:** Moritz Reuter, Edwin Joseph, Guoping Lian, Dominique J. Lunter

**Affiliations:** † Department of Pharmaceutical Technology, Faculty of Science, Eberhard Karls Universität Tübingen, Auf der Morgenstelle 8, 72076 Tuebingen, Germany; ‡ Department of Chemical and Process Engineering, 3660 University of Surrey, Guildford GU27XH, U.K.; § Unilever R&D Colworth, Unilever, Sharnbrook MK441LQ, U.K.

**Keywords:** Stratum Corneum, Molecular Dynamics Simulations, Ceramides, Skin Barrier Function, Skin Permeability, Transepidermal Water Loss

## Abstract

Ceramides, as major components of the human stratum corneum’s (SC) lipid matrix, are considered crucial for regulating the skin’s barrier function against the ingress of exogenous substances as well as to prevent water loss through the skin. Multiple clinical and experimental studies found different classes of ceramide species to affect the skin barrier nonuniformly, with some ceramides being associated with an impaired skin barrier, such as ceramide NS, while others are associated with a healthy, unimpaired skin, e.g., ceramide NP. This study investigates how the presence of these two ceramide classes in an SC lipid bilayer membrane influences the water permeability as well as the structure of the bilayer using molecular dynamics (MD) simulations. To this end, simulated membranes comprising free fatty acids, cholesterol, as well as either ceramide NS or ceramide NP were systematically compared in regard to differences in the membrane structure and water permeability, as well as to results found in the literature. The simulation found ceramide NP-containing membranes to have a significantly lower water permeability than ceramide NS-containing systems, with the permeability values of NP-based systems being almost half of those of the NS-based systems. Furthermore, the simulation also showed significant structural differences between the two systems in terms of headgroup conformation and lipid positioning in the membrane, hinting toward the molecular mechanisms underpinning the differences in permeability of the two systems. In conclusion, the MD simulation was able to reproduce effects of the presence of different ceramide species in the membrane that are consistent with experimental as well as clinical studies on skin barrier function and drug delivery and validate previous simulation-based investigations into SC lipid bilayer permeability.

## Graphical abstract

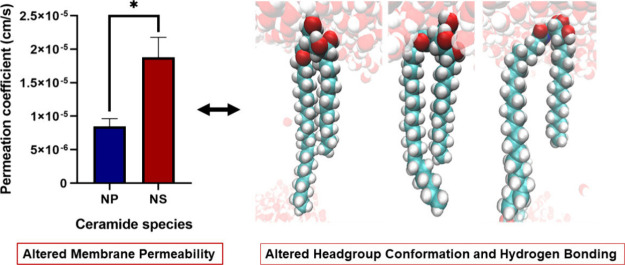

## Introduction

Understanding the barrier function of the stratum corneum (SC), the uppermost layer of the skin, carries high relevance for a variety of biomedical and skin care applications, from the pathology of inflammatory skin diseases to the drug delivery of dermally applied active pharmaceutical ingredients as well as cosmetic care products.^1–4^ As such, there have been an increasing number of investigations aiming to describe, model, and predict the behavior, structure, and underlying mechanisms of the skin’s most vital barrier as well as its interactions with a wide range of topically applied compounds.^4–11^ Integral to this barrier function of the SC is the brick-and-mortar structure, with the keratin-rich corneocytes (bricks) being embedded in a lamellar, continuous lipid matrix (mortar).^12^ While the corneocytes provide a vital role in the protection against UV damage and mechanical stress, the lipid matrix guards against the ingress of xenobiotics as well as water loss from within, acting as the primary diffusion barrier of the skin.^13^ The SC lipid matrix is comprised of equimolar parts of ceramides, free fatty acids (FFAs), and cholesterol,^12^ forming characteristic periodic, lamellar structures, known as the short periodicity phase (SPP), which closely resembles a traditional bilayer structure, and the long periodicity phase (LPP), whose structure, depending on the exact structural model proposed, resembles a sandwiched multilayer structure.^14,15^ The formation and structural characteristics of these lamellar structures have been shown to be highly dependent on the exact composition of the ceramide/FFA/cholesterol ratio, wherein a change in the ratio of the lipid components as well as the subtype of the lipid in question affect the properties of the entire system.^16–22^ Higher ratios of cholesterol in the lipid composition have been associated with an increased fluidization of the bilayer system,^16^ while for FFA and ceramides, pure or near-pure systems show high crystallinity and very low permeabilities in simulated systems.^23,24^ Ceramides, in particular, can be further classified into families, depending on the headgroup type of the long chain base (LCB) moiety as well as the hydroxylation pattern of the fatty acid linked to the LCB.^25–27^ Based on the current state of skin research, more than 16 different ceramide families have been found in human SC, with more than 1300 individual ceramide species when taking fatty acid as well as LCB chain length distribution into account.^27^ Changes in the prevalence of certain lipid subtypes, especially chain lengths, have been associated with changes in the permeability in *in silico* studies investigating the effect of varying chain lengths on the fatty acid moiety of ceramides. The works by Gupta et al.^23^ as well as Wang and Klauda^28^ found a decrease in permeability of ceramide-containing bilayers with longer chain lengths, explained partly due to a higher membrane thickness. Critically, chain length distribution of FFA and ceramides also affects the skin barrier permeability *in vitro* and *in vivo*. Skolova et al.^29^ found the permeability of SC lipid membranes to be decreasing with increasing ceramide acyl chain length, while Janssens et al.^30^ as well as Ishikawa^31^ and others associated shifts toward shorter ceramide acyl chain lengths as well as shorter FFA lengths in live human SC with higher skin barrier permeability, completing the picture of the permeability-decreasing effect of skin lipid chain lengths from *in silico* to *in vivo* studies. Regarding the effects of the relative prevalence of different ceramide families, this picture looks different. While in the human SC there is a wide range of ceramide families being found, with the 10 most abundant families representing over 99% of all detectable ceramides,^26^ this diversity is often not reflected in MD studies. The majority of publications use the sphingosine-derived ceramide NS species in their simulated skin lipid membranes,^5,23,28^ ^,^ ^32–34^ despite its actual abundance in the extracellular matrix of the human SC being quite limited, at around 5% of all ceramides in the SC lipid matrix.^25–27^ On the other hand, the phytosphingosine-derived ceramide NP (depicted with ceramide NS in Figure 1) is the most abundant ceramide subgroup in the human SC lipid matrix, being more than five times as abundant as ceramide NS.^25–27^ Nonetheless, studies investigating NP-containing systems are scarce in comparison to the ones involving NS-containing systems.^6,35^

**Figure 1:**
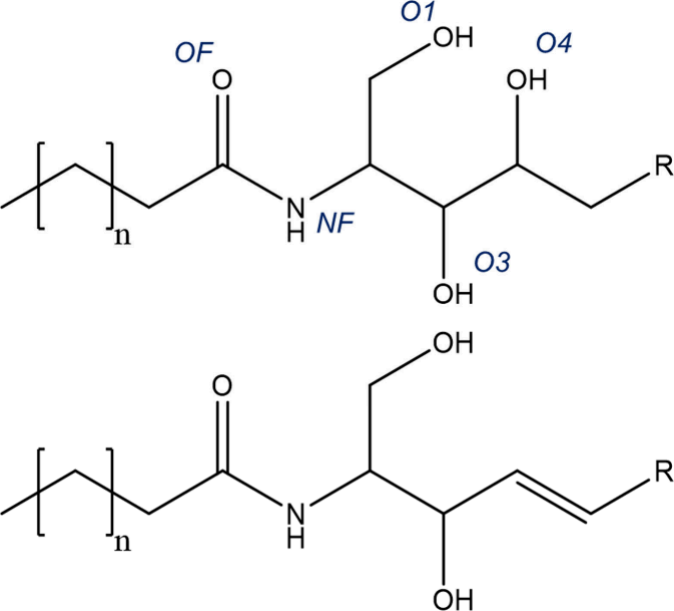
Comparison of the ceramide headgroup structure between ceramide NP (top) and ceramide NS (bottom). The heteroatoms of the ceramide NP structure are designated with their abbreviations used throughout the text.

From a clinical perspective, these ceramide subgroups are of special interest, as the ratio of ceramide NS to NP seems to play a vital role in the development of many skin conditions involving skin barrier impairment.^3,31,36,37^ In particular, the ratio of NS to NP is shifted toward NS in inflamed or damaged skin with an impaired skin barrier, as one would find in atopic dermatitis or psoriasis patients as well as in surfactant-treated skin. Conversely, the ratio is shifted toward NP in intact and healthy skin.^2,31,36,38^ Thus, this begs the question of whether there exists a mechanistic explanation for the different permeabilities of the skin barrier containing different ratios of ceramide species: Nădăban et al. published an in-depth comparison of SPP models of skin lipid mixture with ceramide ratios of 2:1 and 1:2 of NS:NP in which they found pronounced differences in the hydrogen bonding network of the two systems, while the models proved to be quite similar otherwise.^39^ The group did not publish permeability data for the models, but there do exist permeability studies for SC lipid membrane models of the SPP using ceramide NP as well as NS. While most works investigated the permeability of SPP models comprising ceramide NS,^23,28,32,33,40^ Lundborg et al. reported a study on the permeability of water in an SPP model comprising ceramide NP.^6^ Regarding SPP models comprising ceramide NS, Piasentin et al. assembled and compared the assorted literature data regarding permeabilities of NS-containing SPP models.^33^ Taking the data from Lundborg et al. as well as from Piasentin et al., one finds a permeability coefficient of 1.1 × 10^–5^ cm/s for NP-containing and 2.10 × 10^–5^ cm/s for NS-containing fully hydrated bilayers.^6,33^ These data show a lower permeability of NP-containing membranes, as one would expect when hypothesizing a possible mechanistic background for the macroscopic effect of a lower skin barrier function and a higher permeability of ceramide NS-rich skin found in the clinical studies. However, a direct comparison is difficult, as the reported simulations were conducted using different methods (steered vs restrained water) as well as different force fields. While both studies based their simulations on the CHARMM36 force field, Lundborg et al.^6^ modified the force field to more closely match the crystalline structure of ceramide NP, making the comparison to the values reported for NS-containing membranes reported by Piasentin et al.^33^ more difficult.

Therefore, this study aims to systematically compare the two SC lipid bilayer systems containing either ceramide NS or ceramide NP as the ceramide component of the lipid mixture, using the same force field as well as the same method for determining the permeability for both systems. As such, the barrier properties are characterized as the permeability of water through the membrane using the constrained water permeation method as published by Piasentin et al.,^33^ while using the modified force field by Lundborg et al.^6^ for all simulations.

Furthermore, we aim to investigate the structural and mechanistic differences between the two membrane models to gain greater insight into the effects of the presence of different ceramide species in the SC as well as to compare our findings to the literature results for *in silico* as well as experimental studies in order to further close the gap between clinical data and theoretical calculation.

## Materials and Methods

### Generation of the Hydrated Bilayer Systems

To model the SC lipid matrix, hydrated bilayers of the SC lipids were constructed using a lipid ratio of FFA: Cholesterol: Ceramide at 1:1:1, wherein the FFA component is represented by lignoceric acid (C24) and the ceramide component either by ceramide NS 24:0/18:1 or by ceramide NP 24:0/18:0. The lipid bilayer membrane is surrounded on both sides with water molecules, representing a hydration level of 30 water molecules per lipid. Both constructed systems are similar to those established and studied in the literature for investigations of SC lipid membranes.^6,33^ The system configurations were generated using the membrane builder^41^ included in CHARMM-GUI,^42,43^ comprising 75 lipids randomly distributed in each bilayer leaflet in a rectangular geometry, resulting in a system of 50 cholesterol, 50 FFA, and 50 ceramide molecules in the lipid bilayer and 4500 water molecules surrounding it.

All simulations were run in GROMACS 2023.4.^44,45^ The CHARMM36 force field was modified as described by Lundborg et al.^6,46,47^ and used for the main simulations. The reason for the choice of this force field over the unmodified CHARMM36 ^46,48^ force field lies in the inclusion of ceramide NP into the simulated bilayer systems: As shown by Lundborg et al., ceramide NP is not correctly described by the unmodified CHARMM36 force field, requiring modifications to model the headgroup more accurately.^6^ As such, we used the modified CHARMM36 force field, which uses the corrected headgroup geometry for ceramide NP and also adopts the applicable changes to the headgroup geometry for ceramide NS for the simulations of both systems containing the different ceramide species.^6^ Nonetheless, the results of the permeability measurements using the unmodified CHARMM36 force field can be found in Supplementary Figure S2 in the Supporting Information. The water model used was CHARMM36 TIP3P.^49^

For each bilayer system containing a different ceramide species, the starting configuration underwent energy minimization and was equilibrated in two 2.5 ns simulation runs under NVT conditions, followed by a 2.5 ns and three 5 ns equilibration runs under NPT conditions, each with continuously weakening position restraints of the lipids, with the last NPT equilibration run being unconstrained, all according to the standard CHARMM-GUI protocol.^41–43^ Afterward, an unconstrained simulation run under NPT conditions with a duration of 300 ns was carried out for the lipids to assume a stable configuration, and the final configuration of this run was used as a base for the permeability measurement simulations as well as the production runs for the structure analysis.

All simulations use a time step of 2 fs and three-dimensional periodic boundary conditions. Electrostatic interactions are calculated using PME,^50^ while for the van der Waals interactions a cutoff at 1.2 nm with a smooth force-switch from 1 to 1.2 nm was used for calculation. H-bonds are constrained using the LINCS algorithm.^51^ For equilibration as well as production simulations, temperature is set to 303.15 K using a V-rescale thermostat^52^ with a time constant of 1 ps, coupled separately to lipids and water. Pressure in the systems is set to 1 bar with a semi-isotropic C-rescale barostat^53^ with a time constant of 5 ps. A compressibility of 4.5 × 1 × 10^–5^ bar^–1^ is used.

### Production Runs of the Equilibrated Systems

To obtain structural information about the unmodified systems without inserted water molecules, one 200 ns production run was executed for each equilibrated bilayer system using the same run parameters as the previous 300 ns NPT equilibration run. The trajectories of the production runs form the base for the calculation of the bilayer properties, hydrogen bonding values, and lipid order parameters.

### Permeability Calculations Using Constrained Water Molecules

The simulations, measurement, and calculation methods using constrained molecular dynamics simulations for the permeability of the SC lipid systems are carried out according to the method described by Piasentin et al.^33^ The aim of this method is to sample local ΔG as well as the diffusion coefficient D along the entire length of the trans-bilayer axis (*z*-axis), using constrained water molecules inserted into the system to obtain a profile of individual ΔG­(z) and D­(z) values, describing the entire membrane and calculating its permeability using the inhomogeneous solubility diffusion model.

Starting from the final configuration of the unconstrained 300 ns simulation runs of the SC lipid systems, water molecules were inserted into the systems: The length of the reaction coordinate (RC), defined as the part of the *z*-axis to be sampled, was 9 nm. This length allows for sampling of the entire membrane as well as part of the water box surrounding it. Along the reaction coordinate, water molecules were inserted at regularly spaced intervals, “windows”, of 0.1 nm, resulting in 90 equally spaced windows to sample the entire reaction coordinate. While their positions along the *z*-axis of the system were fixed, the positions of the inserted water molecules on the x-,y-plane were random. As to not distort the measurements by having the constrained water molecules potentially interacting with each other while also saving simulation time by not building a new configuration for every position window, the sampling was done using a total of 60 configurations each containing 6 water molecules spaced at regular intervals of 1.5 nm, ensuring no interaction of the constrained water molecules with each other as well as sampling the entire bilayer four times.^54^

Water molecules were inserted using the GROMACS tool “gmx insert-molecules” with a scaling of 0.25, meaning that the van-der-Waals radius of the inserted molecule was reduced to 25% of its original radius to insert the molecule in question for easier insertion into the system as well as a broader insertion range. To not disrupt the systems by abruptly inserting molecules into the configuration, the inserted water molecules were faded in by carrying out four sets of energy minimization/NVT/NPT simulations with a duration of 50 ps each with the electrostatics and van-der-Waals interactions gradually turning on using the coupling constant λ = 0.25/0.5/075/1, wherein λ = 1 represents regular interactions. Decoupling of the molecular interactions was accomplished by using the GROMACS functionalities for free energy calculations. The equilibration simulation runs with partially decoupled interactions utilized a stochastic dynamics integrator and with a reduced time step of 1 fs as well as time constant for thermostat coupling of 2 ps, as recommended by the GROMACS manual. From the equilibration simulations with λ = 1 onward, the regular leapfrog integrator was used again. All inserted water molecules were constrained to a fixed z-position relative to the bilayer center, realized using the GROMACS “pull” command with the “pull-coord-type” set to “constraint”. After the last 50 ps of equilibration with full interactions restored, the resulting systems then underwent a longer equilibration run of 100 ns. Following these equilibration runs, production runs started.

The production run of every 60 configurations lasted for 40 ns, during which the z-component of the force acting on the center of mass of the inserted water molecules as well as the z-position of the inserted water molecules were collected at every time step of the simulation (0.002 ps) while the coordinates of the entire system were stored in every 10 000 timesteps (20 ps).

To determine the permeability *P* for water across the investigated skin lipid membranes, the inhomogeneous solubility diffusion model was employed:^55–57^

$$\frac{1}{P}=\int_{-L/2}^{L/2}\frac{\exp\left[\frac{1}{RT}\Delta G\left(z\right)\right]}{D_{z}\left(z\right)}dz$$

In which L is the thickness of the bilayer, R is the universal gas constant, and wherein the free enthalpy of diffusion ΔG­(z) as well as the diffusion coefficient in the *z*-direction *D*_*z*_(*z*) were calculated as follows:

ΔG was calculated using the Potential of mean constraint force (PMcF) method according to the equation

$$\Delta G\left(z_{0}\right)=-\int_{z_{out}}^{z_{0}}F{\left(z\right)}_{t}\mathrm{dz}$$

wherein ⟨F­(z)⟩_*t*_ is the average of the force acting on the COM of the inserted water molecule averaged over the time of the simulation.^55,56^ The force average was taken from every constrained water molecule in the set of simulations, yielding force averages along the entire RC, starting from bulk water and along the entire transmembrane axis. These values were then binned according to their 0.1 nm window along the RC, and averages as well as standard deviations and errors were calculated. ΔG was then determined via eq 1 by integration of the resulting curve using the trapezoidal rule. The error on the ΔG was derived by error propagation from the variance in the binned force average. To reduce the noise in the PMcF measurements associated with the first few nanoseconds of the run as well as to ensure robust sampling, only data from the last 20 ns of the production run was used for the permeability calculation.^10,33^ To obtain the final ΔG profiles for the permeability calculation, the average force profiles over the reaction coordinate were symmetrized and then integrated to average the bilayer leaflet properties and to address membrane inhomogeneities in the sampling process.^10,33^ Unsymmetrized average force profiles as well as unsymmetrized ΔG profiles can be found in the Supporting Information in Supplementary Figures S8 and S4, respectively.

D_*z*_ was calculated from the Kubo relation, using the equation

$$D_{z}\left(z_{0}\right)=\frac{{\left(RT\right)}^{2}}{\int_{0}^{\infty }\partial F\left(z_{0}.t\right)\times \partial F\left(z_{0}.0\right)dt}$$

in which δF­(z_0_, t) = F­(z_0_, t) – ⟨F­(z_0_)⟩_t_ is the autocorrelation function (ACF) of the force acting on the molecule.^56,58,59^

The force ACF was obtained from the GROMACS tool “gmx analyze”, from which the ACF of the first 50 ps of the simulation was determined and integrated using the trapezoidal rule. The time length of 50 ps was determined following the findings of Piasentin et al., according to whom 50 ps sufficed for a full convergence of the force ACF, especially with a force constant k → ∞ with constrained simulations. All force ACFs decreased to less than 5% of their starting values and were, thus, considered converged.

The entire permeability calculation was carried out according to the PMcF/Kubo method described by Piasentin et al.^33^

### Bilayer Properties Analysis

The properties of the bilayers were investigated for the validation of the method and the systems containing different ceramide species were compared. Density calculations for the investigated bilayers were performed using GROMACS’ “gmx density” tool. Membrane thickness was determined from the density peak of the ceramides’ amide nitrogen, and associated errors were obtained by block averaging over 6 Blocks of 50 ns of the production runs. Area per lipid (APL) measurements were calculated from the box vectors of each frame of the production runs as well as the number of lipids in each membrane fold and averaged over time, with the associated errors obtained from the oscillations around the average. Lipid order parameter S_*z*_ was determined using the GROMACS tool “gmx order” and lateral distance between ceramide chains using “gmx distance”. Hydrogen bonding was analyzed using the GROMACS tool “gmx hbond”.

### Statistical Analysis and Visualization Tools

Statistical analysis was carried out using Graphpad Prism 8, and values were, when applicable, tested for significant differences using Student’s two-sided *t* test. Visualizations of the simulations were prepared using VMD,^60^ while graphs are prepared using Microsoft Excel and Graphpad Prism 8 as well as R Statistical Software using the package ggplot2.^61,62^

## Results

### Bilayer Properties

Graphs comparing the lipid density profiles of the modeled systems can be found in Figure 2. The two types of bilayers show very little difference in their structural properties: The thickness of both bilayers, determined as the distance between the nitrogen atoms of the ceramide headgroups of opposing membrane leaflets, was found to be 4.96 ± 0.02 nm for NP-based bilayers as well as 4.96 ± 0.03 nm for NS-based bilayers. These results agree reasonably well with the findings of previous studies of Piasentin et al.^33^ and Mistry & Notman^5^ for hydrated equimolar skin lipid bilayers, who reported a membrane thickness of 4.90 ± 0.04 nm and 4.89 ± 0.04 nm, respectively. The small discrepancy between the previously published results and the slightly larger bilayer thickness found in this study can be explained by the choice of force field: While Piasentin et al. and Mistry & Notman both used the unmodified CHARMM36 force field, this study used the modified CHARMM36 force field as described by Lundborg et al., providing a modified headgroup geometry, which could affect the thickness of the systems. This was further confirmed by our finding that the unmodified CHARMM36 force field produced thinner bilayers (Supplementary Figure S6 in the Supporting Information). Nonetheless, the results obtained for the membranes in this study using the modified force field still much more closely match the other simulated results than the thickness obtained from experiments such as those reported by Školová et al.^63^ and Schmitt et al.,^21^ which was 5.39 nm (No error given) for an equimolar SC lipid membrane containing ceramide NS^63^ and 5.41 ± 0.01 nm for an SC lipid membrane containing ceramide NP and AP,^21^ differing significantly from the simulation’s results, which is unfortunately characteristic for the type of lipid models investigated in this study. Similar to the membrane thickness, the area per lipid (APL) as a membrane density is very similar for both the NS- and NP-containing systems, both having an APL of 0.322 nm^2^ ± 0.002 nm^2^. This value corresponds well to the values found in the literature, wherein both Piasentin et al. and Mistry & Notman found an APL value of 0.325 nm^2^, again using the unmodified CHARMM36 force field. Similar membrane density of the equimolar SC lipid systems was also reported by Wang and Klauda^64^ for equimolar SC lipid systems containing the structurally even more different ceramide NS and ceramide AP. While the increased hydroxylation of phytosphingosine-based ceramides may pose a bigger steric hindrance to tight lipid packing in systems containing an unphysiologically high ceramide content, this effect is seemingly mitigated by an equimolar amount of lignoceric acid and cholesterol, resulting in similar APL values for both systems.

**Figure 2:**
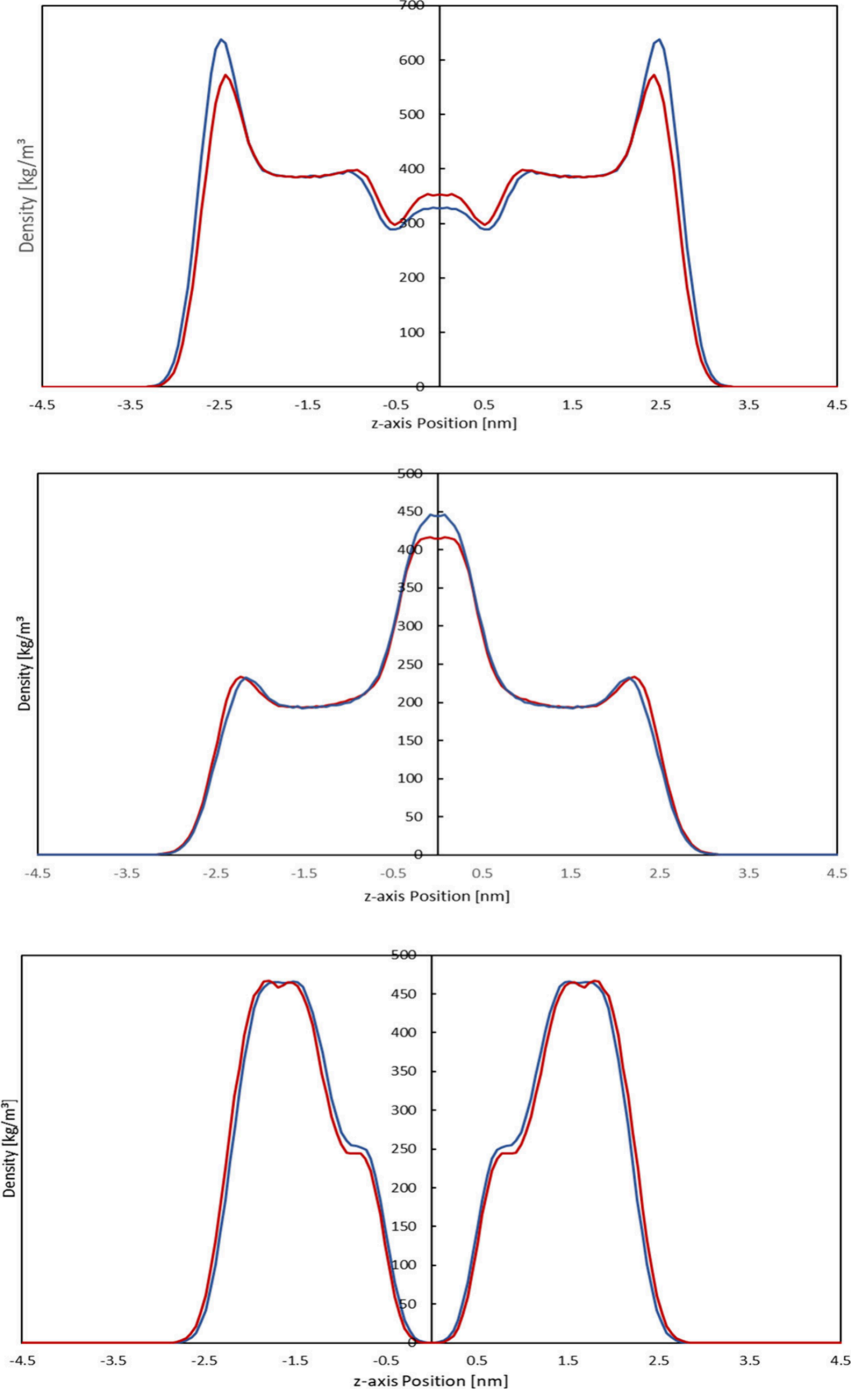
Comparison of the lipid density profiles for ceramides, free fatty acids, and cholesterol (from top to bottom) of SC lipid bilayers containing either ceramide NP (blue) or ceramide NS (red). In systems containing ceramide NP, cholesterol as well as free fatty acids show density peaks closer to the membrane center than in ceramide NS-containing systems, while the density peaks of ceramide NP itself are actually located more distant from the center than those of ceramide NS.

Comparing the density profiles of the systems, one notices slight differences in the locations of the density peaks: In the NP-containing bilayer system, the density curves of cholesterol as well as lignoceric acid are shifted slightly toward the membrane center, resulting in a higher density of lignoceric acid in the membrane center as the interdigitation of the lignoceric acid tails increases. Possible mechanistic explanations for this shift are derived from the hydrogen bonding pattern as well as the headgroup and tail conformations, as described in the following sections.

### Lipid Order Parameters

The probability distribution of the lateral distance of the ceramide species’ lipid chains grants insight into the distribution of possible headgroups as well as chain conformations of ceramides in the hairpin conformer. Wang and Klauda term the possible conformations the “hunched” and “posturing” conformation, with the hunched conformation having a characteristic peak in chain distance at around 0.5 nm, while the posturing ceramide chains are characterized by their peak at a wider distance of more than 0.8 nm.^64^ The probability distributions of the C16 carbon atom’s distances can be found in Figure 3. One peak with a very distinct shoulder at a higher chain separation distance is visible for the systems containing ceramide NP, indicating the presence of both a hunched as well as a posturing conformation. This contrasts to the NS-based systems, which only show a clear peak at around 0.5 nm, with a much closer shoulder at around 0.7 nm, very much favoring the hunched conformer, with the shoulder potentially constituting an intermediate conformation. Interestingly, the probability density distribution of both systems differs from the one found by Wang and Klauda, who did not find a “shoulder” in their distribution but a clear separation of the conformations’ peaks in the distribution curves. We actually found the particular conformation distribution reported by Wang & Klauda^64^ in the system containing ceramide NS simulated using the unmodified force field (Supplementary Figure S10), indicating that the modified force field, which, in particular, modifies the ceramide headgroup geometry, has a profound impact on the conformations adapted by the ceramides in the lipid membranes (For an illustration of the different conformations found in this study, see Figure 4).

**Figure 3:**
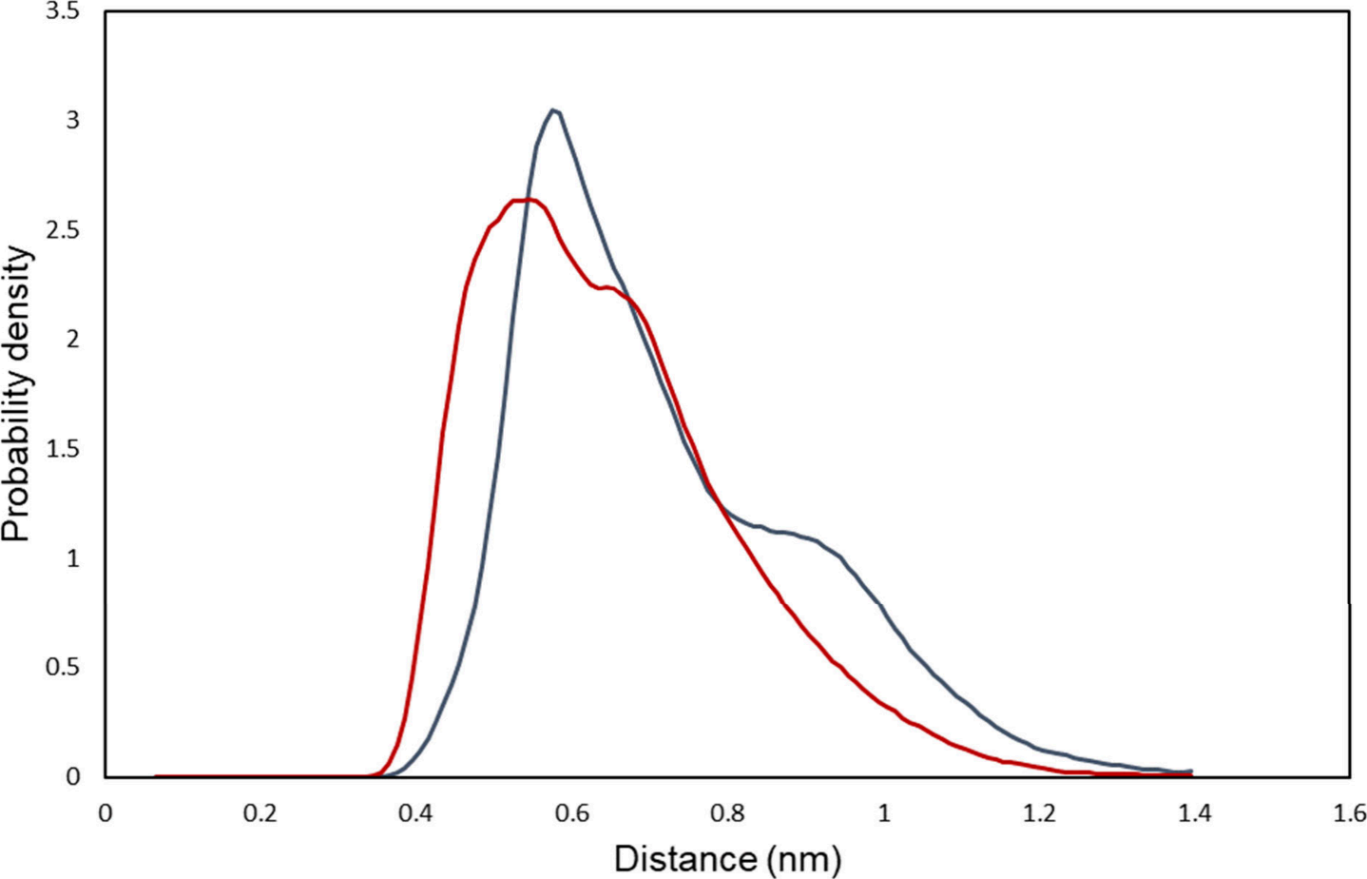
Probability distribution curves (smoothed) of the distance between the C16 atoms of the fatty acid and long chain base moieties of ceramide NP (blue) and NS (red). The probability distributions for the different ceramide types are distinctly different, with NP showing a higher propensity toward the posturing conformation (seen in the shoulder around 1 nm) when compared to NS, whose graph shows a shoulder much closer to the main peak of the hunched conformation, indicating a probable intermediate conformation.

**Figure 4:**
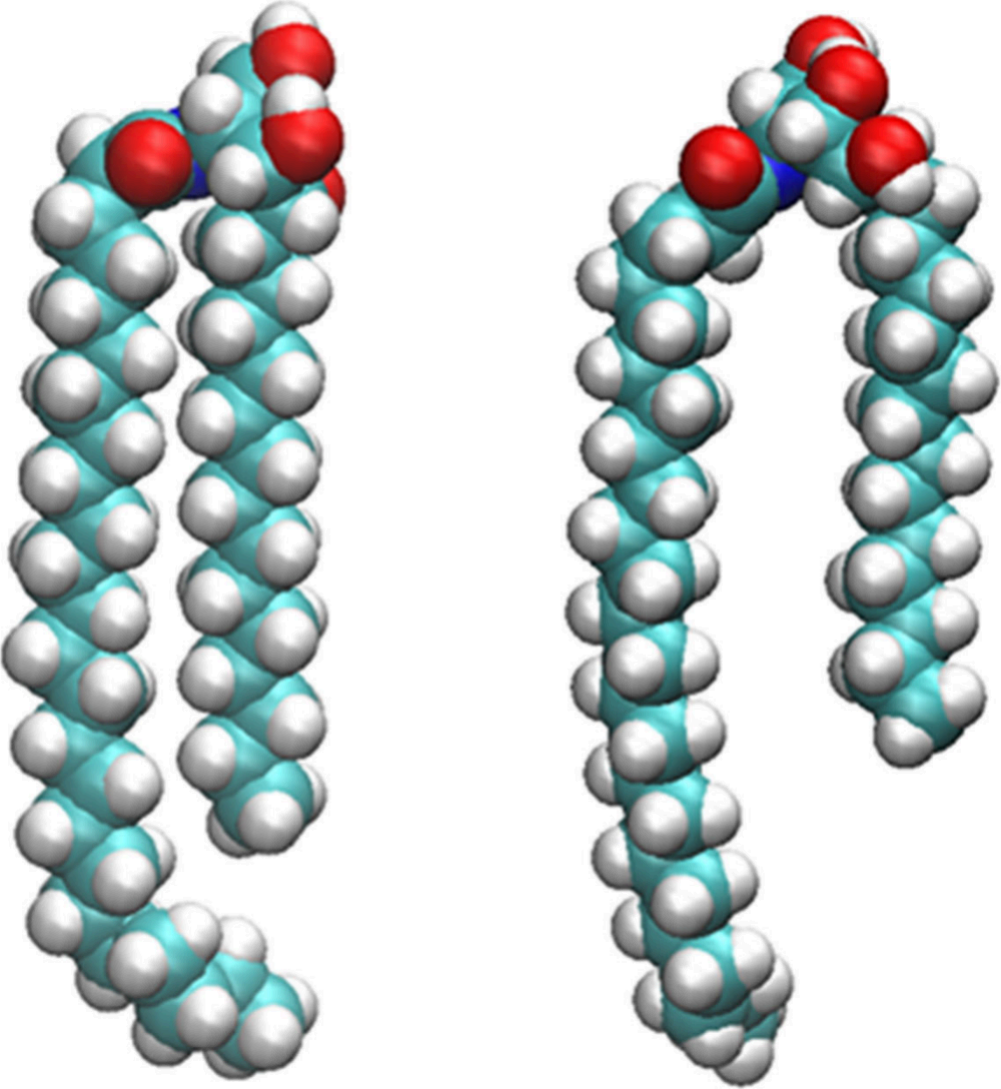
Isolated structures of ceramide NP molecules in either the “hunched” (left) conformation or the “posturing” conformation (right). The posturing conformation is characterized by the higher distance between the two carbon chains of the ceramide as well as the hydroxyl functional groups being rotated toward the membrane normal, therefore being able to interact to a higher degree with the molecules of the bulk water phase. (See Figure 8 and Table 1.) Additionally, due to the higher distance between the carbon chains, cholesterol molecules are able to intercalate between them and potentially hydrogen bond with the amide nitrogen, which can be seen in an increased number of hydrogen bonds between the amide of ceramide NP and cholesterol (Supplementary Table S1).

The lipid order parameter S_*z*_ serves as an indicator for the alignment of a given carbon chain with the *z*-axis of the simulation box normal to the membrane. A higher S_*z*_ value indicates closer alignment with the membrane normal direction, implying a higher degree of lipid order. The S_*z*_ graphs for the lipid tails of the LCB and fatty acid moiety of the ceramides as well as the tail of lignoceric acid are depicted in Figure 5. For both lipid chains of the ceramides, a similar trend can be seen. While the differences are only slight in either case, the S_*z*_ values of ceramide NS show a higher degree of order closer to the headgroup-water interface with ceramide NP showing a higher degree of order toward the center of the bilayer membrane. This observation is consistent with a more prevalent posturing conformation of ceramide NP molecules. In this conformation, the carbon atoms in the lipid tails closer to the headgroup are less parallel to the membrane normal (Figure 4). Furthermore, the order parameter curve for lignoceric acid does not show the same pattern, with only a slight increase in the S_*z*_ value in the NP-containing systems toward the center of the membrane, further hinting toward a conformational difference of the simulated lipid membranes containing different ceramide species.

**Figure 5:**
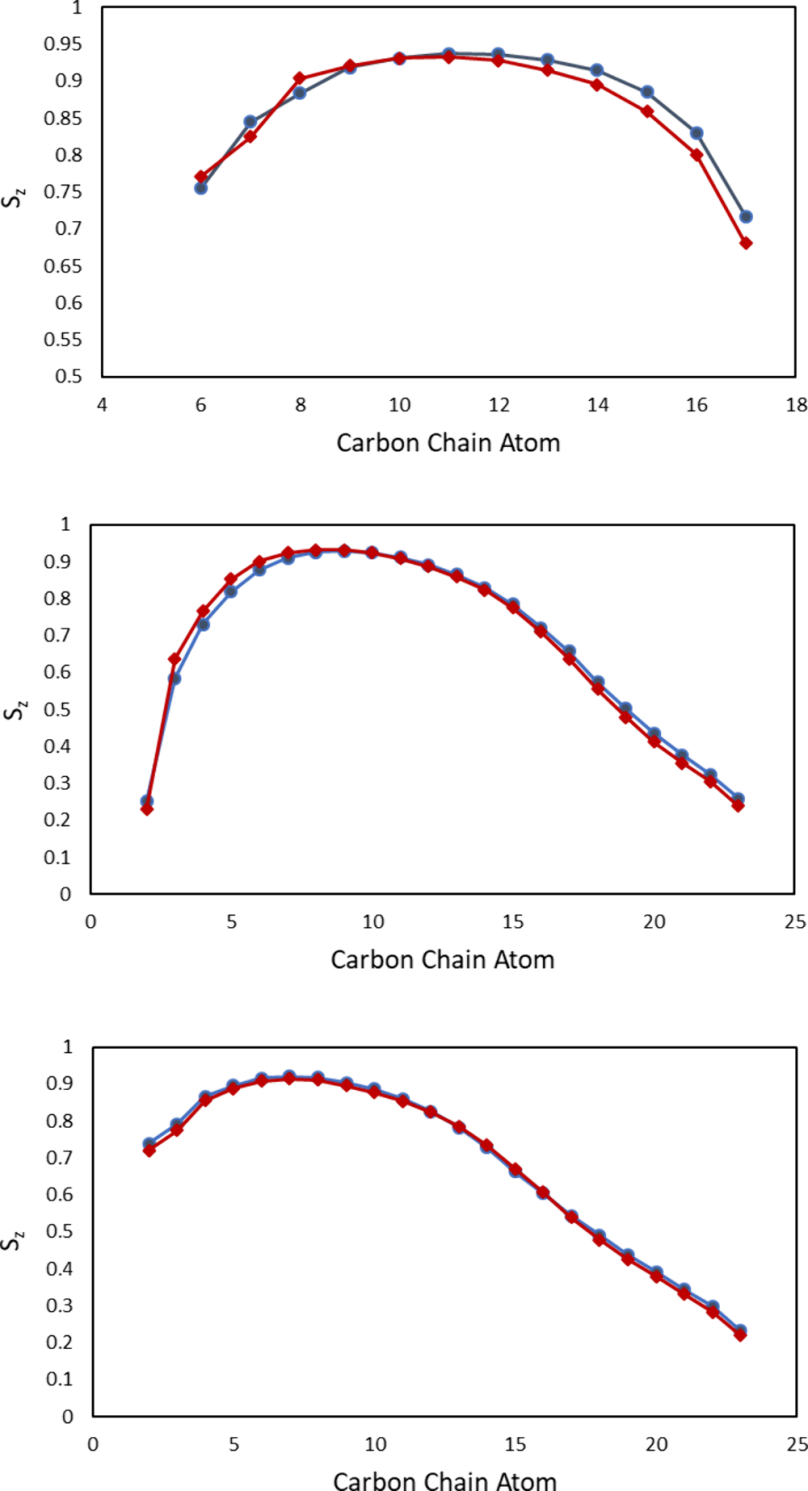
S_*z*_ lipid order parameter curves for carbon chains of the ceramide long chain bases, the fatty acids of the ceramides, and the free fatty acids (from top to bottom, Ceramide NP – blue, Ceramide NS – red). For the carbon chains pertaining to the ceramides, the lipid order is slightly lower for ceramide NP closer to the headgroup, while being higher closer to the membrane center. For the free fatty acids, the curves are essentially the same. Note the different scaling of the uppermost curve.

### Hydrogen Bonding

The type and number of hydrogen bonds found in the simulated systems are provided in Table 1 and Supplementary Table S1.

**Table 1:** Overview of the Hydrogen Bonds for All Lipids in the System, as well as the Ceramides, in Particular^a^

Hydrogen Bond Type	Average number of hydrogen bonds per time frame per lipid (NP/NS)
All Lipids – Water	2.637 ± 0.006/2.407 ± 0.005
All Lipids – All Lipids	0.478 ± 0.003/0.555 ± 0.002
CER – Water	4.322 ± 0.002/3.607 ± 0.002
CER – Other Lipids	1.393 ± 0.001/1.620 ± 0.001

a A more extensive overview of the hydrogen bonding can be found in the Supplementary Material.

The types of hydrogen bonds found in this system can roughly be classified into lipid–lipid hydrogen bonds as well as lipid–water hydrogen bonds, both of which differ between the studied membranes depending on the ceramide class occurring in the bilayer. We found that the NP-based systems show a higher degree of lipid–water hydrogen bonding and a lower degree of lipid–lipid hydrogen bonding. This is a curious result, as one would expect a higher degree of hydrogen bonding in general from the ceramide NP-based systems, given the additional hydroxyl group that it possesses over ceramide NS. A more in-depth analysis of the individual bonding molecules shows actually less hydrogen bonding of the ceramide molecules, in particular, to lipid molecules such as other ceramide as well as lignoceric acid or cholesterol molecules. In terms of lipid–lipid hydrogen bonding in general, only the hydrogen bonding of lignoceric acid with itself is slightly elevated in NP-containing systems. On the other hand, the difference in hydrogen bonding between ceramide NP and bulk water accounts roughly for the entire difference in lipid–water hydrogen bonds of the systems, with ceramide NP exhibiting a distinctively higher degree of hydrogen bonding with the water molecules outside the bilayer than ceramide NS. These results can be understood in the context of a propensity to different conformations by different ceramide species (see lipid order parameters and Figure 3): The posturing conformation is more frequently adopted by ceramide NP molecules, orienting the hydroxyl groups toward the bulk water phase (Figure 9) and facilitating lipid–water hydrogen bonding. On the other hand, the hydroxyl functions in the hunched conformation are turned toward the membrane, facilitating lipid–lipid hydrogen bonding.

### Permeability, PMcF, and Diffusion Coefficient Calculations

The measured water permeability of the ceramide NP-containing bilayer is found to be significantly lower from that of the NS-containing bilayer, with the calculations for the permeation of a water molecule through the membrane returning a permeability of approximately (0.85 ± 0.12) × 10^5^ cm/s for the NP-based SC lipid membrane and a permeability of approximately (1.88 ± 0.30) × 10^5^ cm/s for the NS-based SC lipid membrane, as can be seen graphically in Figure 6.

**Figure 6:**
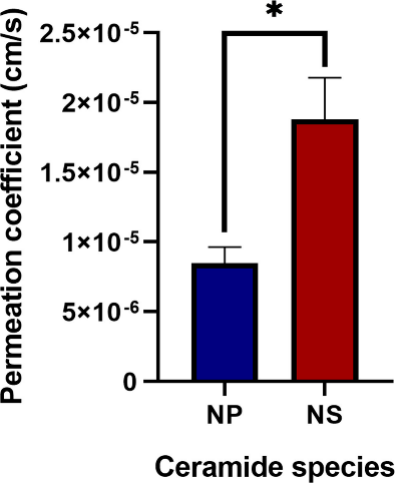
Bar graph of the measured permeation coefficients of the model systems. Ceramide NP-containing systems show a significantly lower permeability than ceramide NS-containing systems. Error bars indicate the standard error; an asterisk denotes a statistically significant difference with *p* < 0.05.

For further comparison, the PMcF profile is shown in Figure 7. The NP-containing bilayer’s PMcF curve shows higher maxima at around 1.5 nm from the bilayer center, as well as tapering off to a higher level toward the membrane center. From this, it becomes clear that the difference in the permeability of the membranes is rooted mainly in the PMcF profile. The maxima of the PMcF most likely correspond to the ordered region of the membrane where not only the long chains of the lignoceric acid and the fatty acid moiety of the ceramides can be found but also the shorter chain of the LCBs. Interestingly, the maxima of the PMcF are shifted around 1 Å toward the membrane center in NP-containing membranes. This could be caused by the sterically more demanding additional hydroxyl group of ceramide NP shifting the region of the densest chain packing toward the membrane center, whereas the *trans*-configured double bond of ceramide NS in this place may be less obstructive to an ordered lipid packing, together with the inward shift of the lignoceric acid as well as the cholesterol density curve as seen in Figure 2.

**Figure 7:**
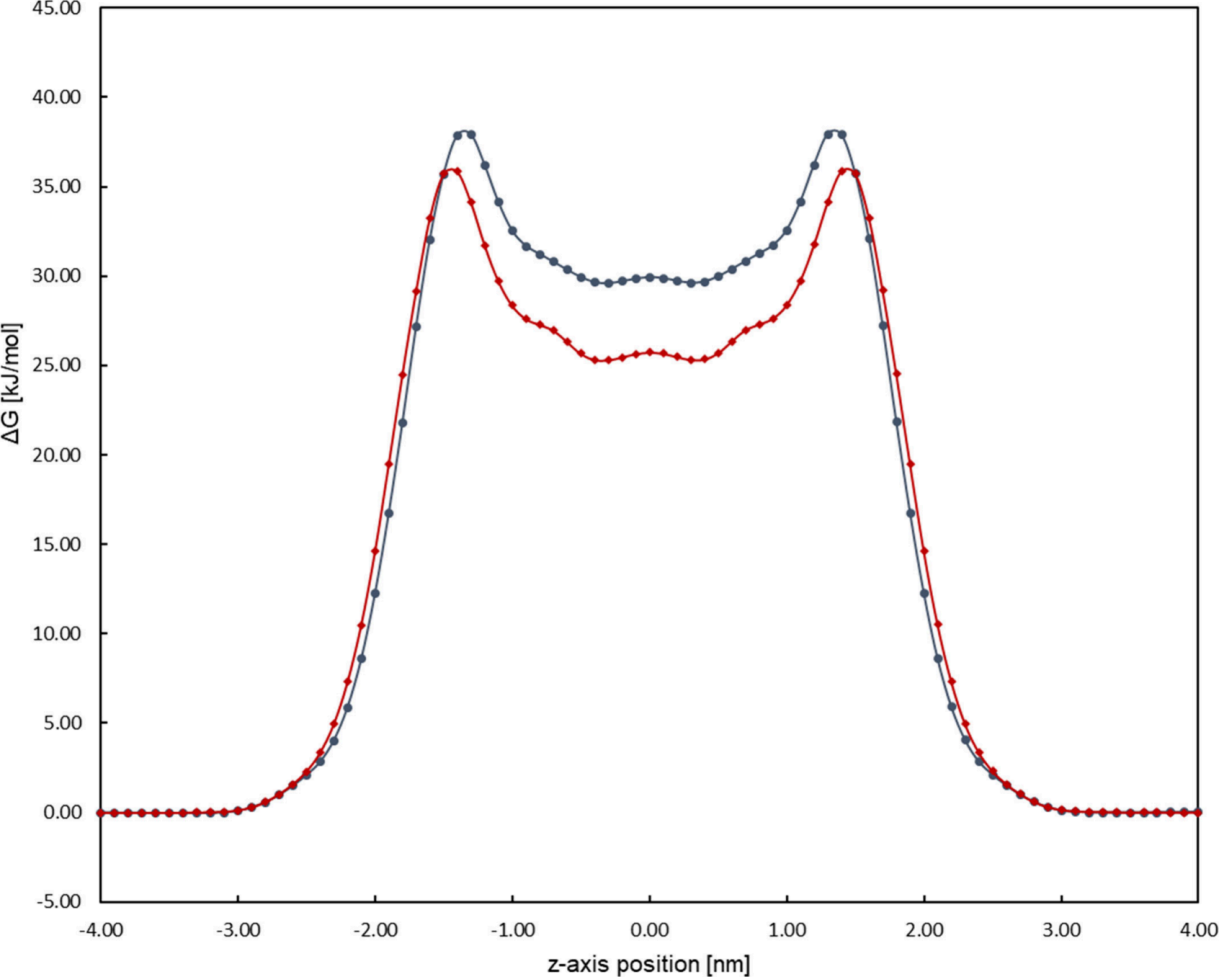
Symmetrized PMcF profile of the measured systems, as used for the permeability calculation. Ceramide NP (blue) shows higher values for the maximum PMcF in the area of ordered lipid tails as well as in the membrane center than ceramide NS (red). Error bars are omitted for clarity (The individual force profiles of the systems with associated error bars can be found in the Supporting Information).

The diffusion profiles D_*z*_ along the bilayer can be found in Figure 8 for both investigated membrane types. The diffusion coefficient profile along the membrane is very similar for both investigated systems, showing almost no significant difference between the two, which contrasts with the measured PMcF profile, which shows quite clear differences between the ΔG­(z) derived from the two model systems. This indicates that the main difference in the resulting permeability values of the two systems stems from the differences in the PMcF profiles, as opposed to the D_*z*_ measurements.

**Figure 8:**
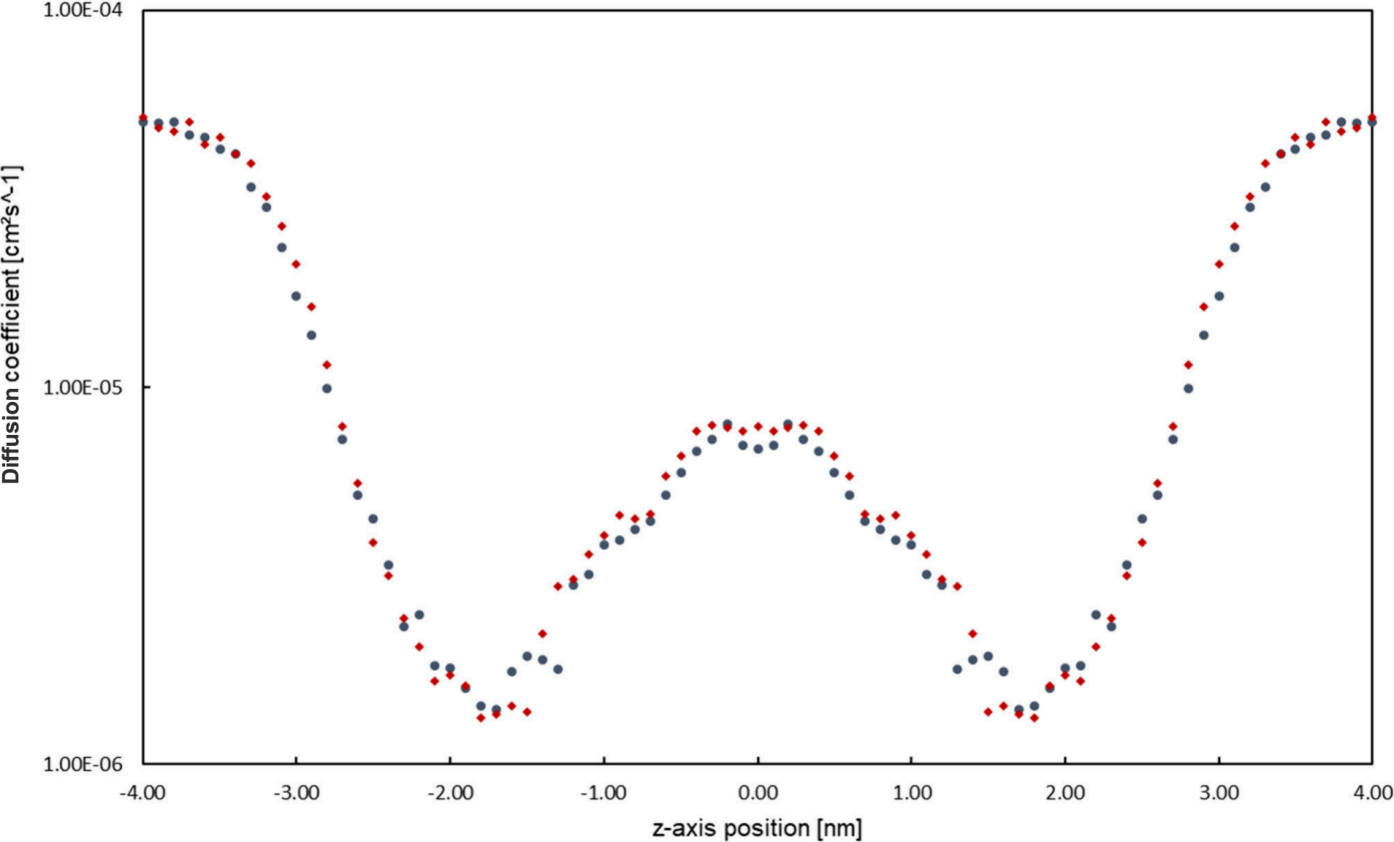
Symmetrized D_*z*_ profile of the measured systems (Ceramide NP – Blue and Ceramide NS – Red). Note the logarithmic scaling of the *y*-axis (Unsymmetrized profiles as well as the individual profiles with associated error bars can be found in the Supporting Information).

## Discussion

The SC lipid matrix, and in particular its role as a permeation barrier for pharmaceutical ingredients, exogeneous chemicals, and endogeneous water, has been the subject of more and more theoretical investigations in terms of the underlying mechanisms and the critical molecular parameters influencing them.^5,7,10,11^ ^,^ ^23,32,65,66^ While these studies yielded valuable insights into the mechanisms and effects of chemical penetration enhancers as well as the effect of varying the concentration, hydroxylation, and chain length of the SC lipids on the membrane,^11,23,32,64^ studies investigating the effect of ceramide species on the permeability of SC lipid membranes are limited. We therefore investigated the effects of varying the ceramide species in SC lipid bilayers on their permeabilities by comparing SC lipid membranes comprising either ceramide NS or ceramide NP as the ceramide component of the system. We found a significantly lower water permeability of the studied ceramide NP-based membrane when compared to that of the ceramide NS-membrane, with the calculated permeability values of NS-containing membranes being almost double the permeability of NP-containing membranes. Although a direct comparison between the clinical and experimental findings and the results we obtained from the simulations is difficult, our simulation results are in qualitative agreement with previous studies investigating the effects of the ceramide NS:NP ratio on the skin barrier function in human skin as well as in experimental models:^31,36,39,67^ Clinically, skin barrier impairments in inflammatory skin diseases are associated with higher levels of ceramide NS and lower levels of NP in the SC lipids and an increased transepidermal water loss (TEWL).^31,36,38^ Similarly, Nădăban et al. experimentally found a significantly higher TEWL value for skin lipid models *in vitro* containing mainly ceramide NS over those models containing mainly ceramide NP. With regard to other investigations utilizing MD simulation methodology, the higher permeability of ceramide NS containing membranes is also in good agreement with previous findings: Lundborg et al.^6^ reported a permeability of (1.1 ± 0.23) × 10^–5^ cm/s for NP-containing membranes, while for NS-containing membranes, Piasentin et al.^33^ reported a permeability of (2.10 ± 0.39) × 10^–5^, almost twice as high as the one found by Lundborg et al.^6^

Yet, it is important to note how this investigation differs from the mentioned works. The investigation by Lundborg et al.^6^ used steered MD simulations, while in this work, we used the constrained MD simulation method described by Piasentin et al.^33^ On the other hand, Piasentin et al. used the unmodified CHARMM36 force field,^46,48^ whereas we used the CHARMM36 force field modified by Lundborg et al.^6^ due to its more accurate representation of ceramide NP in the membrane (See the Supporting Information for a comparison using the unmodified force fields). Taking this into account, our work validates these previous findings by conducting simulations that directly compare SC lipid membranes containing ceramide NS or ceramide NP using the same force field as well as the same method of permeability measurement for both systems. As such, our results show that the difference in permeability found between these systems can indeed be explained by the choice of ceramide present in the system and not simply due to different force fields or permeability measurement methods.

Thus, the simulations reveal a structural molecular basis underpinning the experimental findings of an increase in the TEWL for ceramide NS-containing systems over ceramide NP-containing systems. The detected differences in permeability may originate from their structures. Despite both systems showing similarities in the overall membrane properties such as the lipid bilayer thickness and lipid order parameters, there still exist differences such as in the density distribution of lignoceric acid and cholesterol. In NP-containing systems, these lipids are shifted slightly toward the center by 0.5 to 1 Å, with this change in localization of lignoceric acid in particular resulting in a shift of the peak of the PMcF curves of the system. The maximum PMcF values can be found in the area of ordered lipid tails, which would be shifted together with the lipids. Note that this effect is exclusive to cholesterol and lignoceric acid: The relative positions of the ceramide headgroups actually show a slight shift away from the center. A possible explanation for the shift of the cholesterol could be the higher inclination of the NP-containing systems toward the posturing conformation as described by Wang and Klauda,^64^ in which the ceramide is able to stabilize the cholesterol molecule below the headgroup in between the spaced lipid tails. This agrees with the observation of a higher degree of hydrogen bonding between the amide group and cholesterol found in NP-containing systems (Supplementary Table S1). Regarding the lignoceric acid, the additional hydroxyl group O4 of ceramide NP provides a hydrogen bonding site deeper in the membrane than the carbonyl oxygen OF and O3 of ceramide NS, therefore being able to stabilize the fatty acid closer to the membrane center (Figure 2 and Supplementary Table S1). The different conformational propensities of the systems may also explain the differences in the lipid–water hydrogen bonding: When comparing the ceramide NP molecule in the posturing and the ceramide NS molecule in the hunched conformation (Figure 5), the headgroup of the posturing conformation presents its hydroxyl and carbonyl moieties distinctively toward the membrane-water interface. The hydroxyls and carbonyls of the hunched conformation are much more shielded from the bulk water phase water and oriented inward toward the other lipids of the membrane. This would of course facilitate hydrogen bonds between water and ceramides in the posturing conformation, with the hunched conformation favoring lipid–lipid hydrogen bonding with other headgroups at the membrane-water interface. Figure 9 further illustrates this by depicting ceramide NP molecules at the lipid–water interface from a hunched conformation to a posturing conformation, in the process of which the hydroxy groups of the ceramide gradually turn toward the bulk water phase, which is found to be in good agreement with the presented results.

**Figure 9:**
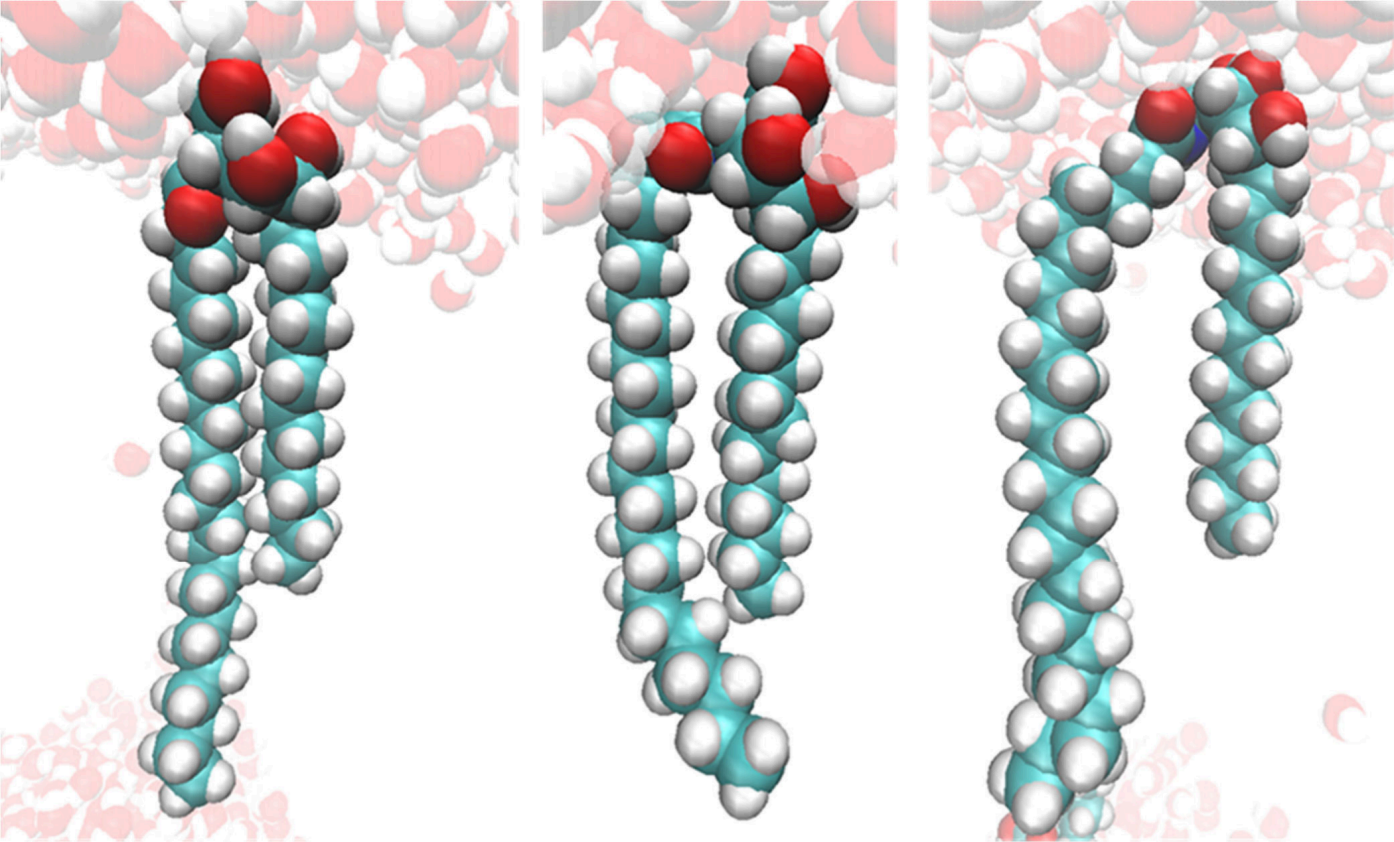
Snapshots of ceramide NP molecules in different conformations interacting with the bulk water phase, from the hunched conformation (left) to the posturing conformation (right), with an intermediate conformation in between. The orientation of the hydroxyl groups toward the water phases is much more pronounced in the posturing conformation, while the hunched conformation’s hydroxyl groups are more oriented toward the lipid membrane.

While this hydrogen bonding pattern (more hydrogen bonds with the bulk water, less hydrogen bonds between lipids) may appear counterintuitive when one takes the permeability results into account, these findings agree with the results of the investigation of Mistry & Notman.^5^ In their study into the mechanisms of permeation enhancer propylene glycol, skin lipid bilayers with a higher permeability for water in the presence of propylene glycol showed more lipid–lipid hydrogen bonding than those with a lower permeability. Furthermore, the only hydrogen bonds whose occurrence was associated with lower permeabilities were lipid–water hydrogen bonds, which mirrors our findings.

While our study was successful in illustrating the differences between SC lipid systems containing different ceramide species, one also has to note its limitations: First, while this study utilized lipid membranes comprising the SC lipids in the equimolar amount as found in human SC lipid matrix, multiple studies hint toward the fraction of cholesterol being overrepresented in simulations using equimolar amounts of lipids, as a significant portion of the cholesterol in the skin may exist in crystalline domains without mixing into the other lipids.^8,16,68^ Second, this study aims primarily to compare the effects of different ceramide species in skin lipid mixtures. Thus, the total replacement of one ceramide species with the other was chosen to be able to observe potential changes more clearly as well as to better compare the results to literature values utilizing only one type of ceramide species in the skin lipid membrane. In reality, the shift from predominantly high ceramide NP concentrations to high ceramide NS concentrations in physiological and diseased stratum corneum is gradual and not absolute.^2,31,36^ To add to this, other lipid species, in particular other ceramide species such as those with hydroxysphingosine or dihydrosphingosine as the LCB or alpha-hydroxylated fatty acid moieties, are present in the SC as well and not represented in this model.^3,31,36^ Lastly, the simulation considered an SC lipid bilayer surrounded by a bulk water, which may only correspond to reality in the outermost layers of the SC lipids in very hydrated skin but otherwise would not be very common. While the SC in living skin is usually hydrated up to 50% of its dry weight, bulk free water domains in the intercellular regions are only present at extremely high hydration levels.^69^ On the other hand, multiple studies have succeeded in modeling systems of the SPP as well as the complex LPP with lower degrees of hydration.^4,6,7,11,67^ The SC lipid membranes with lower degrees of hydration show complex phenomena especially in regard to ceramide and lipid conformations in general, so a follow-up to this study using partially hydrated systems would allow for more insight into the interplay between the constituents of the SC and its barrier function.

## Conclusion

This study investigated the influence of the type of ceramide species present in model bilayers of SC lipids on the permeability as well as the membrane structure: Utilizing constrained MD simulations, we were able to show a significantly lower water permeability of ceramide NP-containing membranes in comparison to that of ceramide NS-containing membranes. Structural investigations into the model systems revealed distinct conformational differences in the ceramide species mostly related to the different headgroups of the ceramides, which, in turn, then further changed the hydrogen bonding patterns and lipid tail conformation of the ceramides in the membrane. Our results agree well with findings from experiments as well as simulations from the literature and enable further investigations into the relation between the SC lipids and its unique diffusion barrier.

## Supplementary Material



## Notes

Render of the bilayer membranes with inserted water molecules; Permeability comparison of systems using the modified and unmodified force fields; Detailed comparison and table of the hydrogen bonding patterns of ceramide NS and NP; APL over time for initial 300 ns runs as well as production 200 ns runs; Density graphs of the ceramide headgroup functional groups (PDF)

## Abbreviations

APL: area per lipid
SC: stratum corneum
PMcF: potential of mean constraint force
COM: center of mass
ACF: autocorrelation function, SPP, short periodicity phase
LPP: long periodicity phase
FFA: free fatty acids
LCB: long-chained base
TEWL: transepidermal water loss
RC: reaction coordinate
NVT: Constant Number, Volume, Temperature
NPT: Constant Number, Pressure, Temperature
