# Supplementary material for: Presence of Different Ceramide Species Modulates Barrier Function and Structure of Stratum Corneum Lipid Membranes: Insights from Molecular Dynamics Simulations
Source: Mol Pharm. 2025 Jun 25;22(7):4280–92. doi: 10.1021/acs.molpharmaceut.5c00580 (PMC12239081; doi:10.1021/acs.molpharmaceut.5c00580)
Supplement: Supplementary file 1 [file mp5c00580_si_001.pdf]

Supplementary Material to “Presence of Different Ceramide Species Modulates Barrier Function and Structure of Stratum Corneum Lipid Membranes: Insights from Molecular Dynamics Simulations”

*Moritz Reuter[1], Edwin Joseph[2], Guoping Lian[2,3], Dominique J. Lunter[1] **

[1] Department of Pharmaceutical Technology, Faculty of Science, Eberhard Karls Universität Tübingen, Auf der Morgenstelle 8, 72076 Tuebingen, Germany

[2] Department of Chemical and Process Engineering, University of Surrey, Guildford GU27XH, U.K.

[3] Unilever R&D Colworth, Unilever, Sharnbrook MK441LQ, U.K.

Table of Contents:

Supplementary Table ST1: Detailed comparison and table of the hydrogen bonding patterns of ceramide NS and NP headgroup with other membrane lipids

Supplementary Figure S1: Ceramide NP-containing model bilayer with and without highlighted inserted water molecules

Supplementary Figure S2: Comparison of the permeabilities of NS/NP-containing systems in unmodified CHARMM36 forcefield and modified CHARM36 forcefields

Supplementary Figure S3: APL over time for the 300 ns initial as well as the 200 ns production simulation runs of both ceramide NS- and NP-containing systems

Supplementary Figure S4: PMcF curves with error bars of NS/NP-containing systems in unmodified CHARMM36 forcefield and modified CHARM36 forcefields

Supplementary Figure S5: Density graph comparison of the ceramide headgroup functional groups

Supplementary Figure S6: Density graph comparison of the ceramide amide nitrogen for determining the membrane thickness

Supplementary Figure S7: Force profiles over time for different locations in the systems

Supplementary Figure S8: Average force profiles over the reaction coordinate of NS/NP-containing systems

Supplementary Figure S9: Diffusion coefficient profiles over the reaction coordinate of NS/NP-containing systems with associated errors

Supplementary Figure S10: Probability distribution curves for comparison of the conformations of NS/NP-containing systems simulated using unmodified CHARMM36 forcefield

References for the supplementary material

Bond Type	Average number of bonds (NP)	Average number of bonds (NS)
Amide - Cholesterol	4.997 ± 0.035	3.548 ± 0.031
Amide - FFA	2.77 ± 0.032	4.283 ± 0.037
O1 - Cholesterol	0.392 ± 0.014	0.583 ± 0.014
O1 - FFA	0.594 ± 0.017	0.36 ± 0.014
O3 - Cholesterol	1.032 ± 0.02	4.28 ± 0.037
O3 - FFA	1.137 ± 0.022	0.991 ± 0.022
O4 - Cholesterol*	1.394 ± 0.025	N/A
O4 - FFA*	3.586 ± 0.038	N/A
OF - Cholesterol	4.874 ± 0.033	6.231 ± 0.037
OF - FFA	4.228 ± 0.032	8.964 ± 0.048

Supplementary Table 1: Average number of hydrogen bonds of the ceramide headgroup functional groups with other membrane lipids per timeframe. The number of hydrogen bonds for ceramide NS outnumbers ceramide NP for almost every type of ceramide-lipid hydrogen bonding, with the notable exceptions of the Amide-Cholesterol bonding as well as the O1-FFA bonding. The increased Amide-Cholesterol bonding for ceramide NP can be seen as a result of the higher prevalence of the posturing conformation as described in the main manuscript, as the posturing conformation allows the cholesterol molecules to intercalate between the ceramide chains and hydrogen bond with the amide (1). In contrast, the hunched conformation, which is more prevalent for ceramide NS molecules, turns the headgroups more toward the membrane and facilitates lipid-lipid hydrogen bonding (see the main manuscript). Bond types marked with an asterisk* are only found in ceramide NP, as ceramide NS misses these functional groups. Nomenclature of the oxygens according to Figure 1 of the main manuscript.

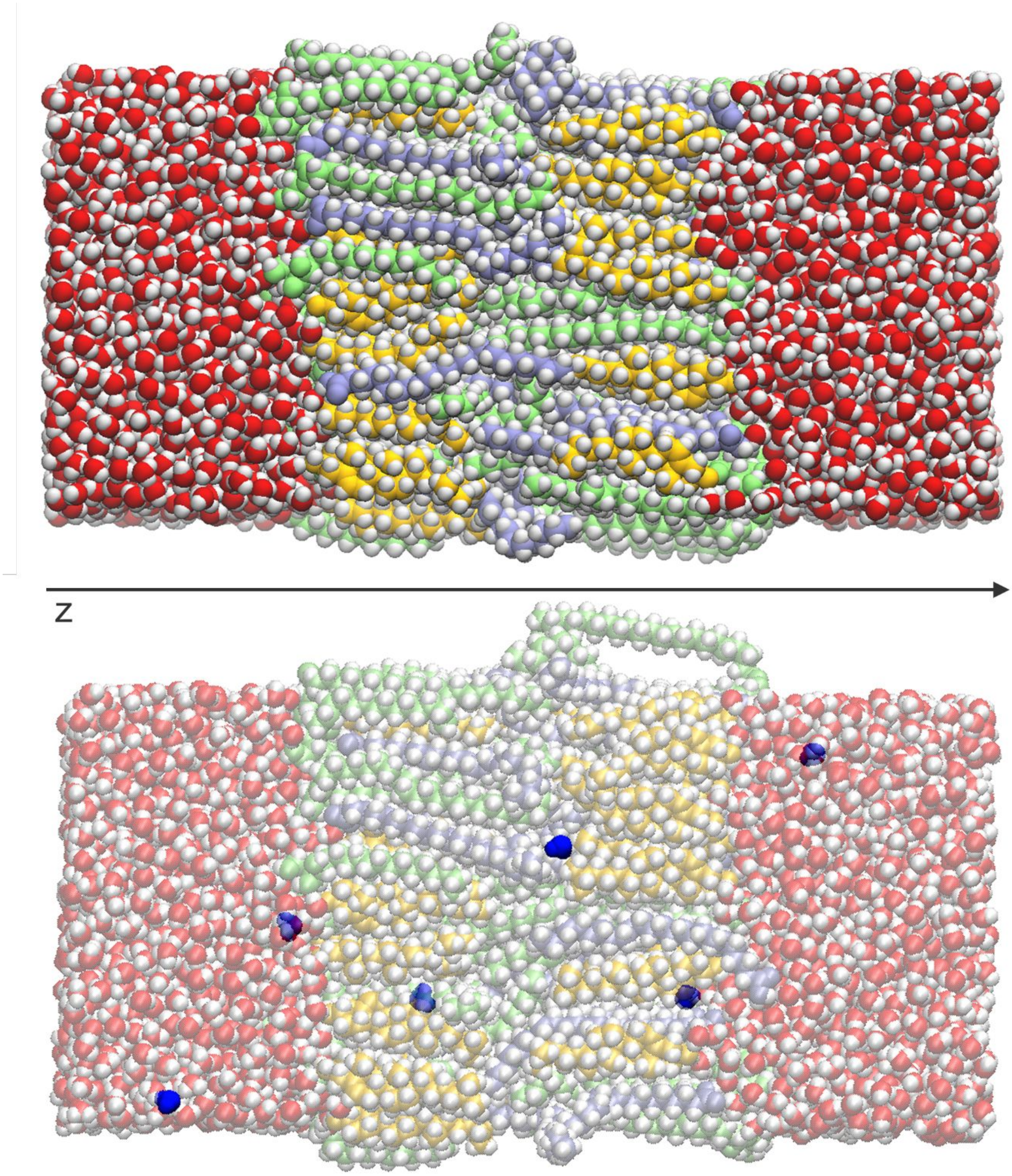

Supplementary Figure S1: Visualization of the last frame of the initial 300 ns run simulating a ceramide NP-based membrane (top) and figure of the bilayer with inserted water molecules for permeability measurement using constrained water molecules (bottom). For the constrained simulations, water molecules were inserted and fixed in relation to the bilayer center at certain positions along the z-axis (as seen in the figure). For each new configuration, the z-axis position for all water molecules was shifted, with a minimum distance of 1.5 nanometer between the molecules kept, and a new run started. (2)

Color Legend: Cholesterol: Orange; Ceramide: Lime; Fatty acid: Ice blue, Bulk water: Red, Inserted water molecules: Blue, hydrogens in white.

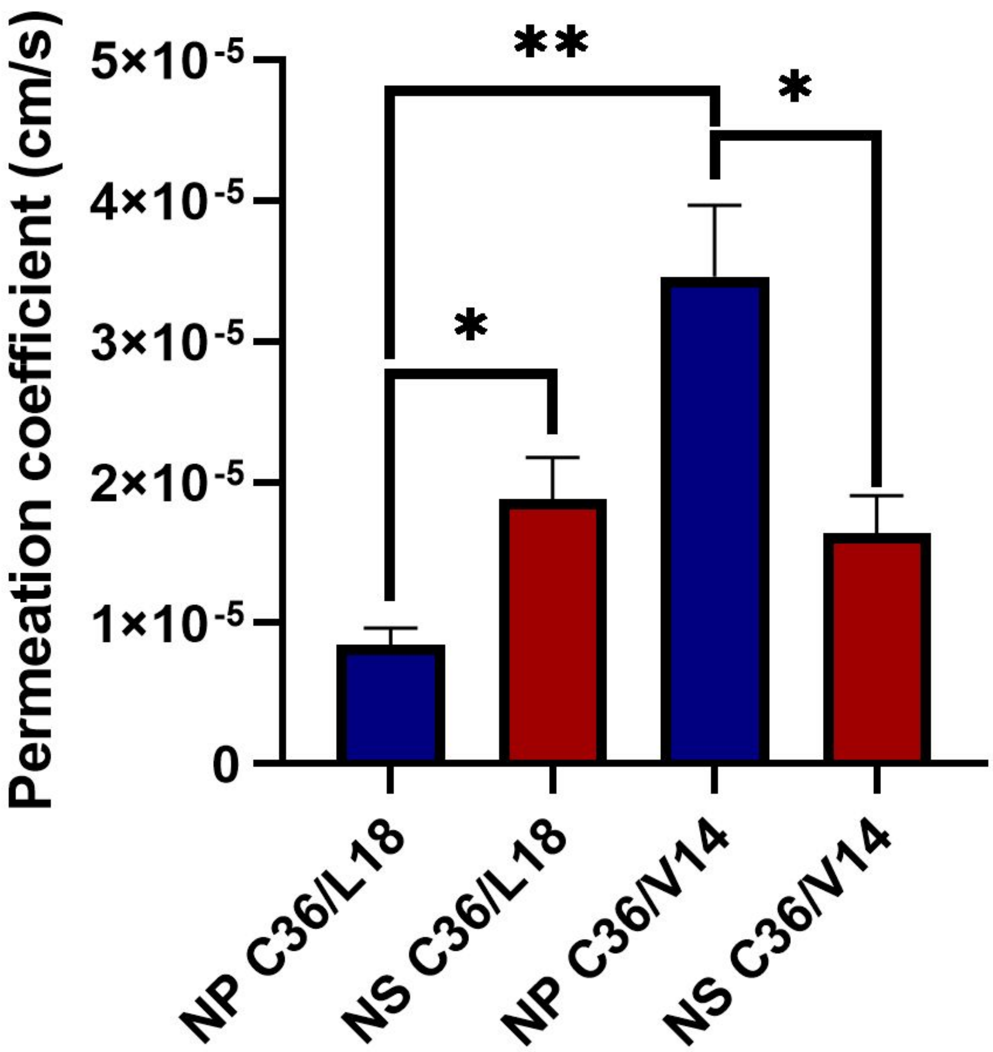

Supplementary Figure S2: Comparison of the calculated permeabilities for NP- and NS-containing systems using either the unmodified CHARMM36 forcefield (3, 4) (C36/V14) or the modified CHARMM36 forcefield (5) (C36/L18). The difference obtained from the different forcefields are significant for NP-containing systems, while the permeabilities calculated for the NS-containing systems are not significantly different from each other. The reason for the discrepancies for the ceramide NP-containing systems is described by Lundborg et al. (5), as the unmodified CHARMM36 forcefield is not able to accurately reproduce the headgroup arrangement of the CER 24:0 NP headgroup crystal structure, which may result in distorted permeabilities in the simulations. Therefore, the revised and modified CHARMM36 forcefield as presented by Lundborg et al. (5) for CER24:0 NS and CER 24:0 NP is presented in the permeability measurements as well as investigated structurally in the main manuscript. One asterisk* denotes a p-value < 0.05, two asterisks** a p-value of < 0.01.

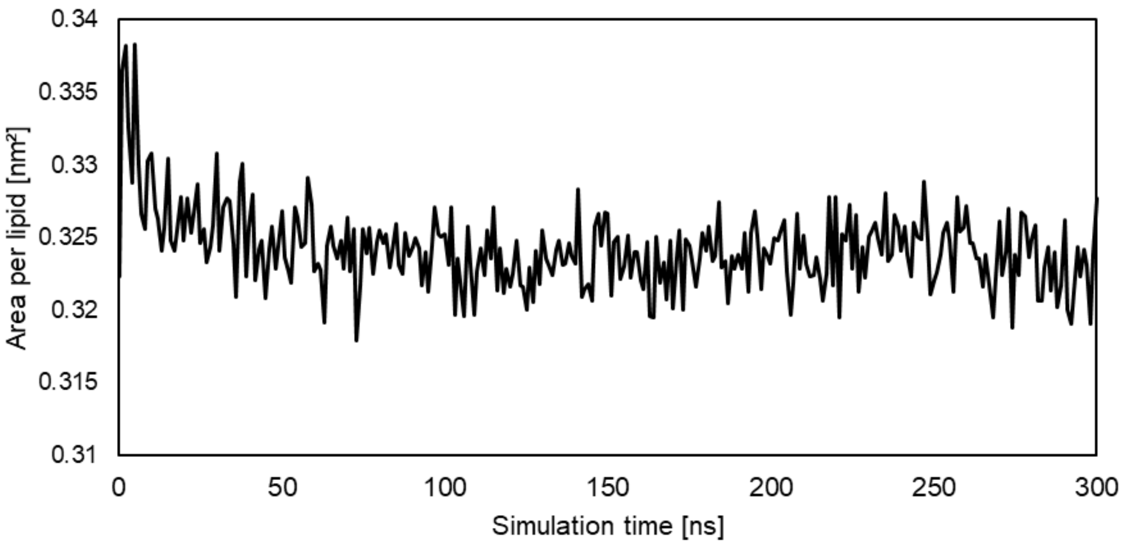

0.34
0.335
0.33
0.325
0.32
0.315
0.31
Area per lipid [nm²]
0
50
100
150
200
250
300
Simulation time [ns]

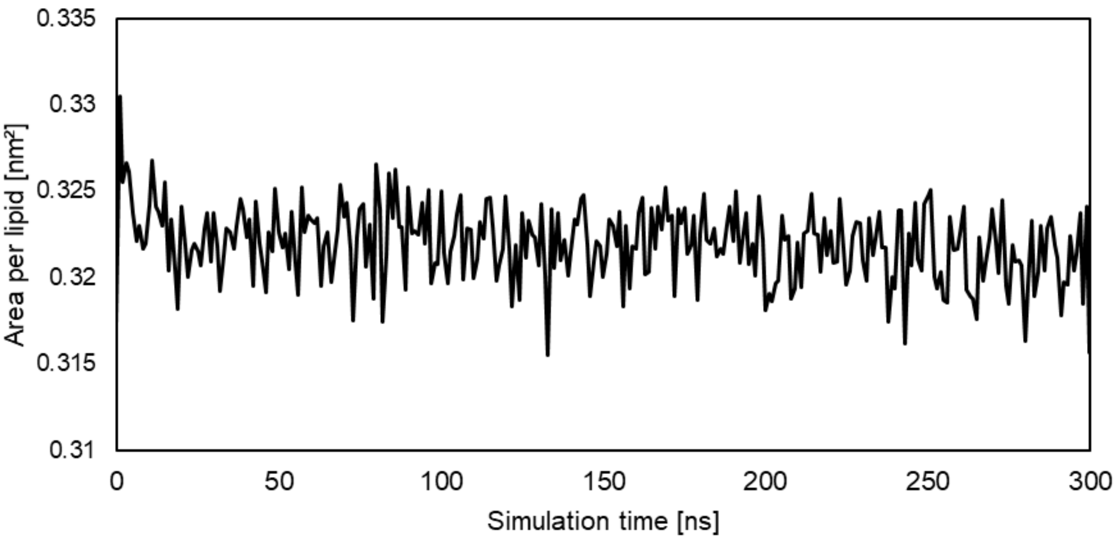

0.335
0.33
0.325
0.32
0.315
0.31
Area per lipid [nm²]
0
50
100
150
200
250
300
Simulation time [ns]

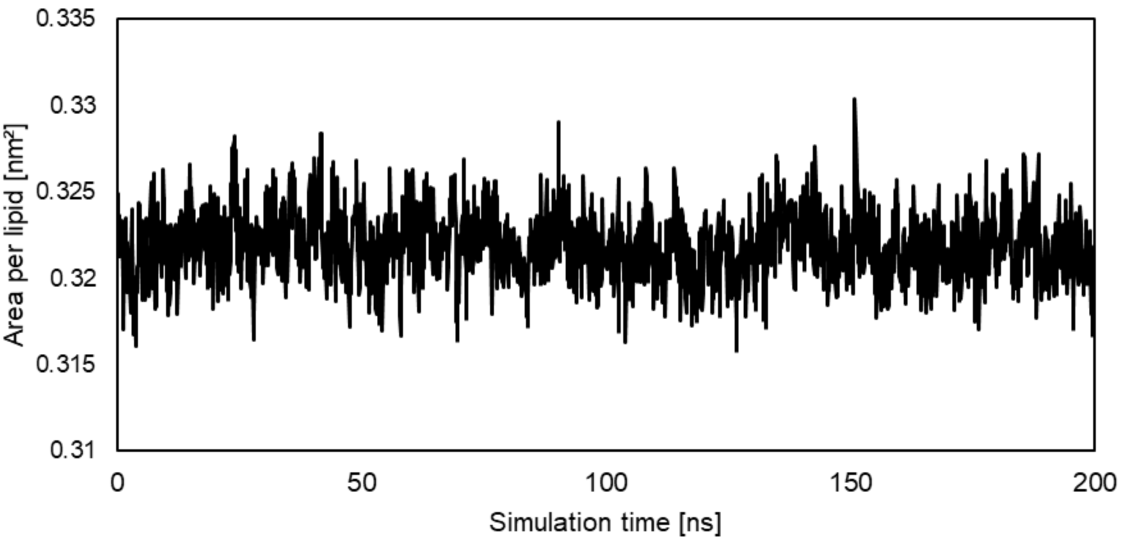

0.335
0.33
0.325
0.32
0.315
0.31
Area per lipid [nm²]
0
50
100
150
200
Simulation time [ns]

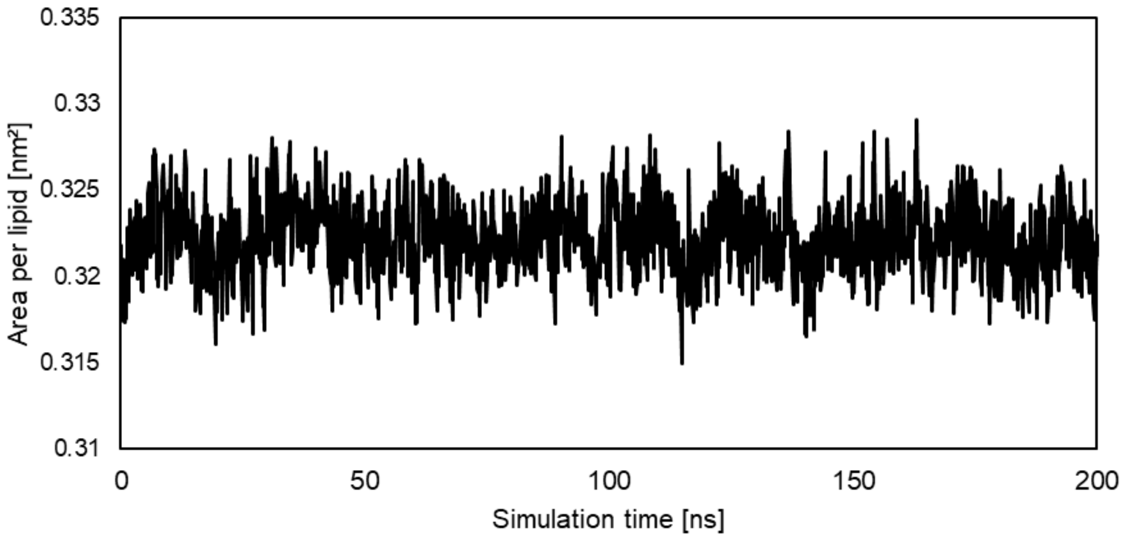

Supplementary Figure S3: APL over time for the initial 300 ns simulation of the ceramide NP-containing system as well as the ceramide NS-containing system, with the APL over time for the 200ns production runs for ceramide NP as well as ceramide NS (from top to bottom). For the equilibration, after an initial drop in APL, the values settle down relatively quickly and fluctuate around a steady value, validating the equilibration time used. During the production runs, the mean APL values fluctuate around a steady value over time as well. For the APL calculation during equilibration, 1 frame every nanosecond was used, while for the production runs, 10 frames per nanosecond were captured.

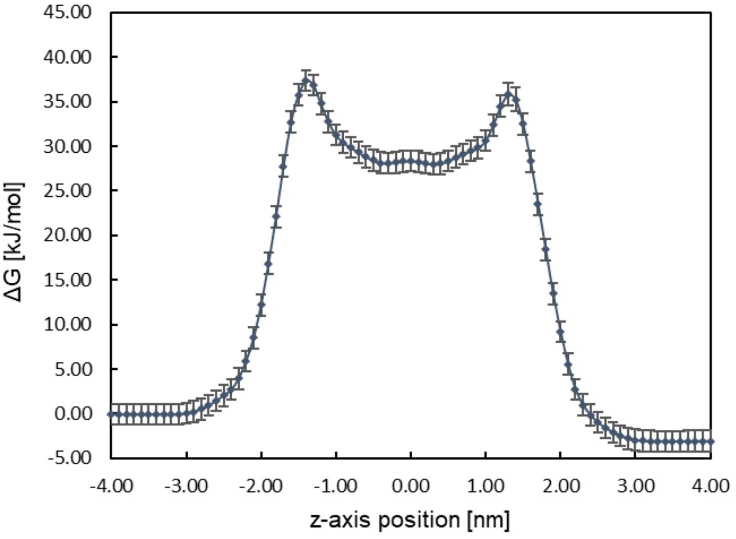

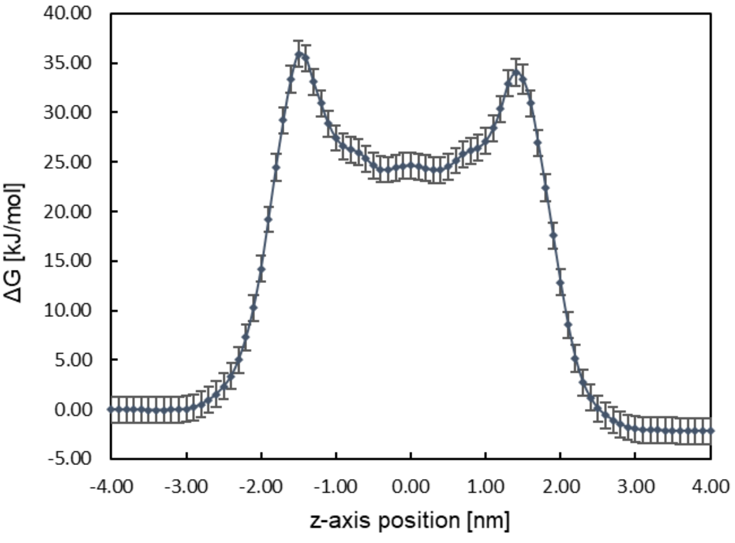

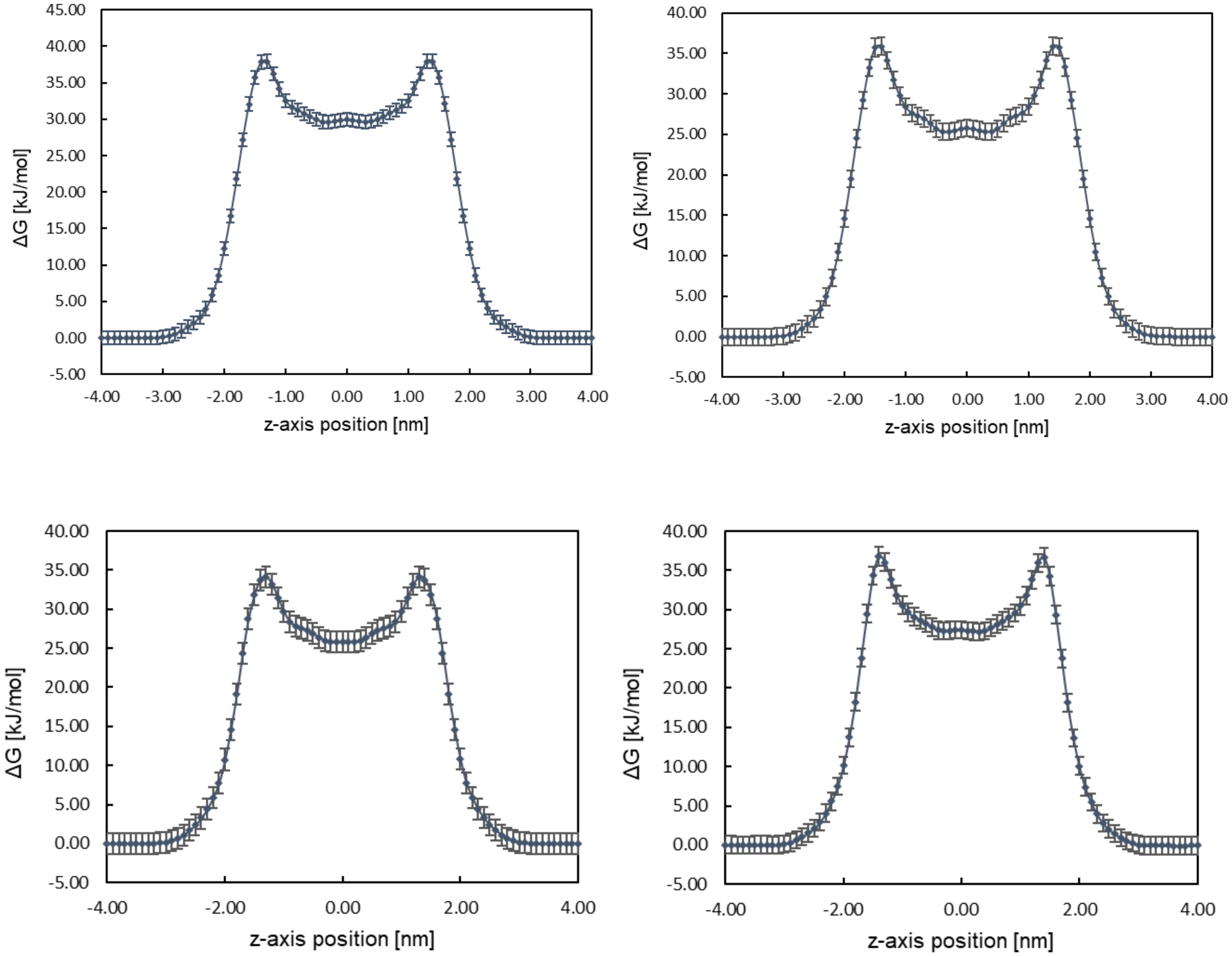

Supplementary Figure S4: Profiles of the PMcF over the membrane with error bars. Profiles of the PMcF before symmetrization are depicted for ceramide NP (top left) and ceramide NS (top right) simulated with the modified CHARMM36 forcefield. The symmetrized profiles used for the permeability calculation are depicted for ceramide NP (center left) and ceramide NS (center right) simulated with the modified CHARMM36 forcefield as well as NP (bottom left) and NS (bottom right) with the unmodified forcefield.

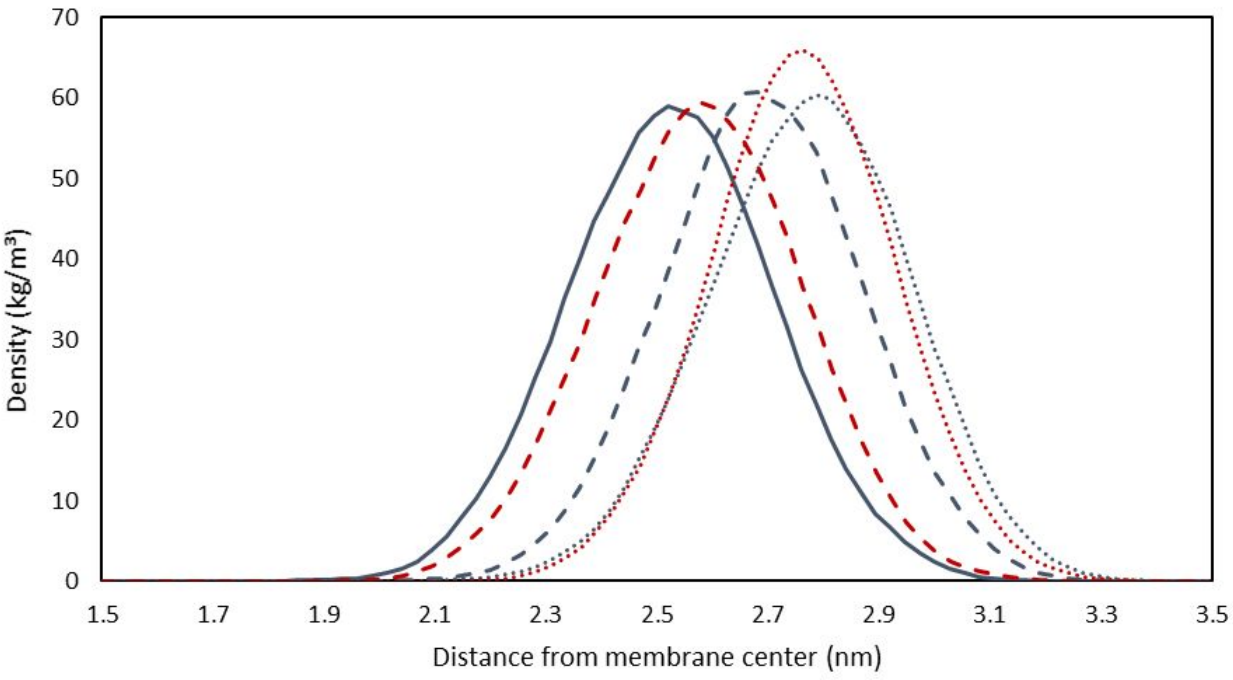

Supplementary Figure S5: Smoothed density graph of the hydroxyl functional groups of the ceramide headgroup. The density peaks of hydroxyl groups O1 and O3 of ceramide NS are located closer to the membrane center than the corresponding density peaks of ceramide NP, which would agree well with the more prevalent hunched headgroup conformation of ceramide NS: As can be seen in Figure 4 of the main manuscript, the hunched conformation positions the headgroup closer towards the membrane than the posturing conformation. Nonetheless, the additional hydroxyl group of ceramide NP is still located deepest in the membrane, providing a hydrogen bonding site especially for FFA (see table ST1).

Legend: Red curves correspond to ceramide NS functional groups, blue curves to ceramide NP. O1: Dotted line, O3: Dashed line, O4: Solid line. Nomenclature according to Figure 1 of the main manuscript.

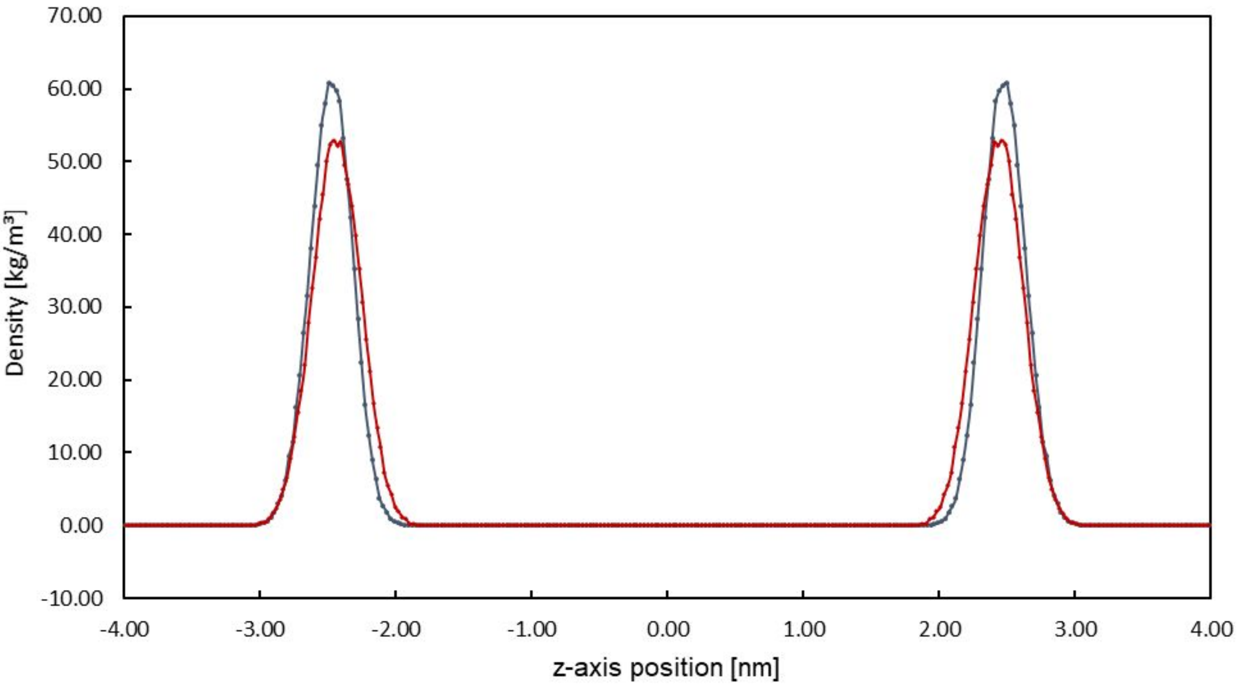

Supplementary Figure S6: Density graph of the amide nitrogens of the ceramide NS-containing systems simulated using the modified CHARMM36 forcefield (blue) as well as the unmodified CHARMM36 forcefield (red). The peaks of the amide nitrogen density curves were used for membrane thickness calculation. The membranes simulated with the unmodified forcefield are slightly thinner than with the modified forcefield.

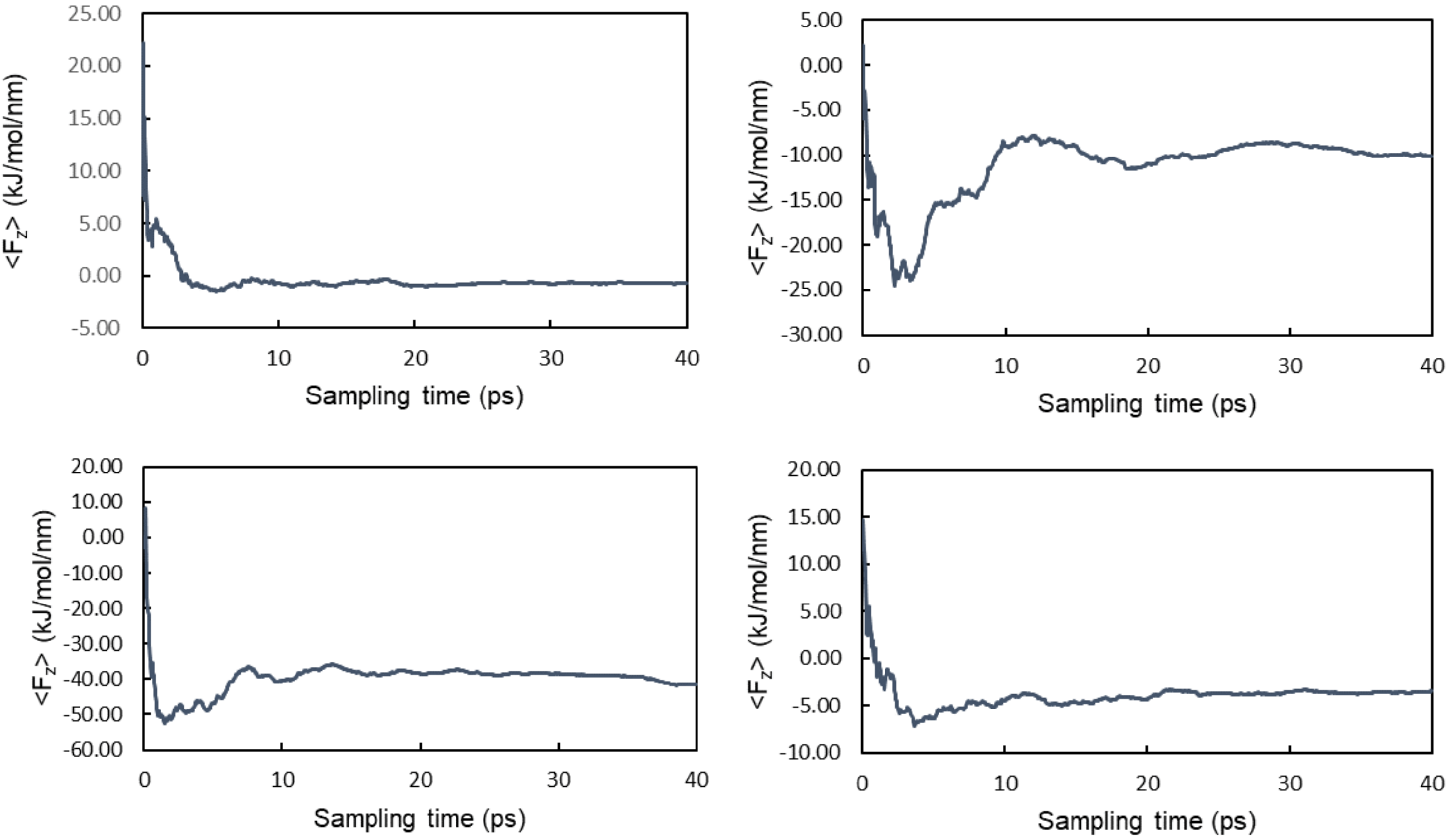

Supplementary Figure S7: Force profiles over time for 4 distinct regions in the bilayer systems (Force profiles obtained from the 40 ns production run of the ceramide NP-containing system). The force profiles depict the force over time for the inserted molecule in the bulk water phase (top left), bilayer headgroup region (top right), ordered lipid tails region (bottom left) and membrane center (bottom right). As can be seen in the graphs, the profiles are very noisy in the first few nanoseconds of the measurement, after which the fluctuations diminish, which is why the average force values for the PMcF permeability calculations were obtained by averaging the last 20 ns of the profiles.

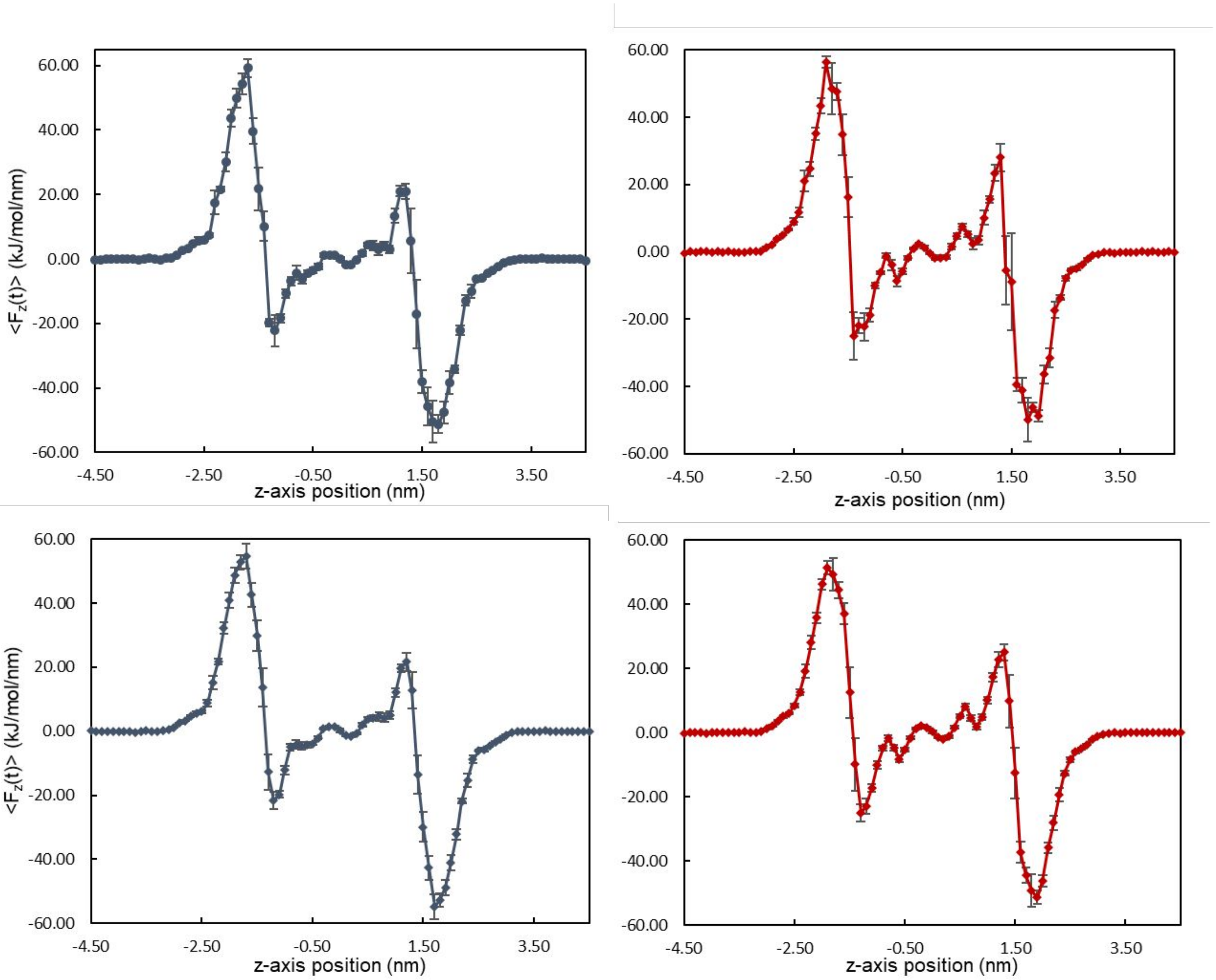

Supplementary Figure S8: Average force profiles over the reaction coordinate. Unsymmetrized profiles for ceramide NP (blue) and ceramide NS (red) can be found in the top row, while the symmetrized profiles can be found in the bottom row.

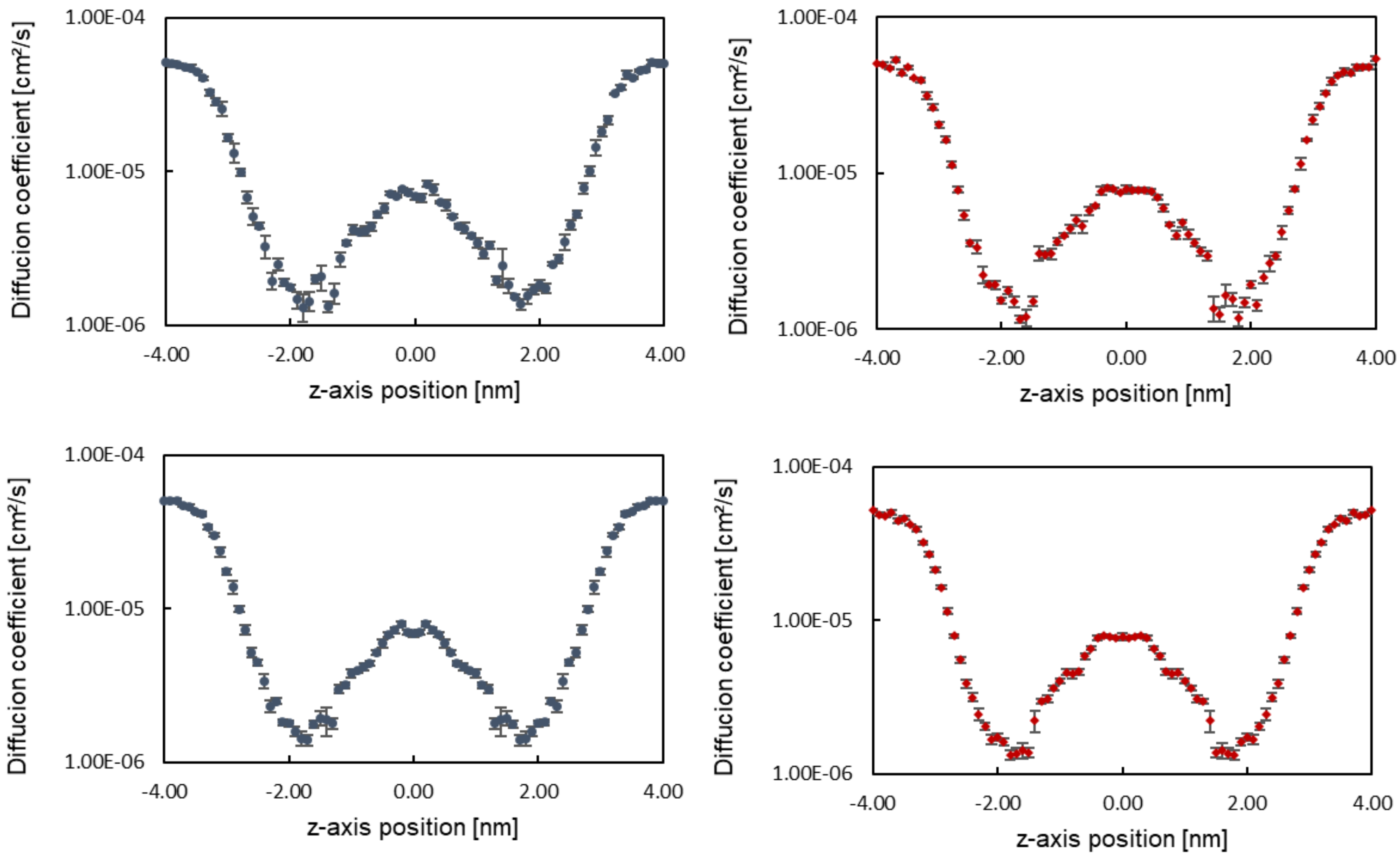

Supplementary Figure S9: Individual local diffusion coefficient profiles along the reaction coordinate. Unsymmetrized profiles for ceramide NP (blue) and ceramide NS (red) can be found in the top row, while the symmetrized profiles can be found in the bottom row. A larger graph comparing the symmetrized profiles without error bars can be found in the main manuscript.

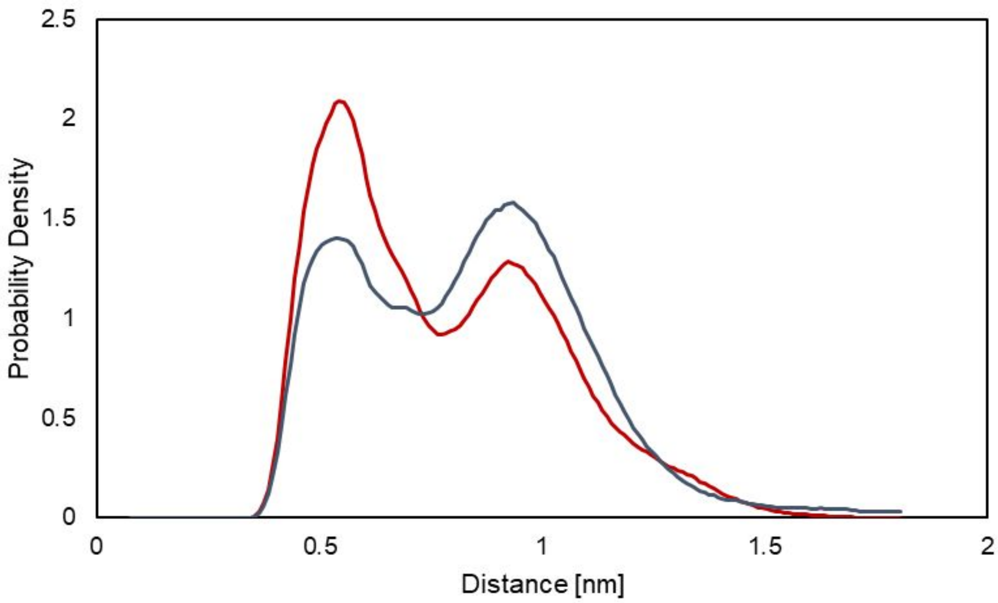

Supplementary Figure S10: Probability distribution curves (smoothed) of the distance between the C16 atoms of the fatty acid and long chained base moieties of ceramide NP (blue) and NS (red) for the systems using the unmodified CHARMM36 forcefield. The probability distribution of the tail-tail distance of ceramide NS agrees well with the findings of the work of Wang & Klauda (1) with a distinct bimodal distribution between the hunched conformation (at shorter distances of ~ 0.5 nm) and the posturing conformation (at ~ 1 nm). The distribution also differs from the probability distribution of the NS-containing system simulated using the modified forcefield presented in the main manuscript, implying that the conformation assumed by the molecules in the system is highly dependent on the headgroup geometry. This is even more clear when one considers the probability distribution of ceramide NP, which shows similarly a bimodal distribution, but one with a highly pronounced shift towards longer distances between the tails. This distribution contrasts with the one obtained from the modified forcefield in the main manuscript as well as with the distributions presented by Wang & Klauda (1). As the unmodified headgroup geometry of ceramide NP was found to not accurately represent the real molecule in membranes (5), the resulting conformation probability distribution may also be distorted and is reported for completeness.

References for the Supplementary Material

1 . Wang E, Klauda JB. Models for the Stratum Corneum Lipid Matrix: Effects of Ceramide Concentration, Ceramide Hydroxylation, and Free Fatty Acid Protonation. The Journal of Physical Chemistry B. 2018;122(50):11996-2008.10.1021/acs.jpcb.8b06188.

2 . Piasentin N, Lian G, Cai Q. Evaluation of Constrained and Restrained Molecular Dynamics Simulation Methods for Predicting Skin Lipid Permeability. ACS Omega. 2021;6(51):35363-74.10.1021/acsomega.1c04684.

3 . Klauda JB, Venable RM, Freites JA, O'Connor JW, Tobias DJ, Mondragon-Ramirez C, et al. Update of the CHARMM All-Atom Additive Force Field for Lipids: Validation on Six Lipid Types. The Journal of Physical Chemistry B. 2010;114(23):7830-43.10.1021/jp101759q.

4 . Venable RM, Sodt AJ, Rogaski B, Rui H, Hatcher E, MacKerell AD, Jr., et al. CHARMM all-atom additive force field for sphingomyelin: elucidation of hydrogen bonding and of positive curvature. Biophys J. 2014;107(1):134-45.10.1016/j.bpj.2014.05.034.

5 . Lundborg M, Narangifard A, Wennberg CL, Lindahl E, Daneholt B, Norlén L. Human skin barrier structure and function analyzed by cryo-EM and molecular dynamics simulation. Journal of Structural Biology. 2018;203(2):149-61.https://doi.org/10.1016/j.jsb.2018.04.005.
